# Gene Expression Profiling and Molecular Characterization of Antimony Resistance in *Leishmania amazonensis*

**DOI:** 10.1371/journal.pntd.0001167

**Published:** 2011-05-24

**Authors:** Rubens L. do Monte-Neto, Adriano C. Coelho, Frédéric Raymond, Danielle Légaré, Jacques Corbeil, Maria N. Melo, Frédéric Frézard, Marc Ouellette

**Affiliations:** 1 Departamento de Fisiologia e Biofísica, Instituto de Ciências Biológicas, Universidade Federal de Minas Gerais, Belo Horizonte, Minas Gerais, Brasil; 2 Centre de Recherche en Infectiologie du Centre de Recherche du CHUL and Département de Microbiologie, Immunologie et Infectiologie, Université Laval, Québec, Québec, Canada; 3 Département de Médecine Moléculaire Faculté de Médecine, Université Laval, Québec, Québec, Canada; 4 Departamento de Parasitologia, Instituto de Ciências Biológicas, Universidade Federal de Minas Gerais, Belo Horizonte, Minas Gerais, Brasil; McGill University, Canada

## Abstract

**Background:**

Drug resistance is a major problem in leishmaniasis chemotherapy. RNA expression profiling using DNA microarrays is a suitable approach to study simultaneous events leading to a drug-resistance phenotype. Genomic analysis has been performed primarily with Old World *Leishmania* species and here we investigate molecular alterations in antimony resistance in the New World species *L. amazonensis*.

**Methods/Principal Findings:**

We selected populations of *L. amazonensis* promastigotes for resistance to antimony by step-wise drug pressure. Gene expression of highly resistant mutants was studied using DNA microarrays. RNA expression profiling of antimony-resistant *L. amazonensis* revealed the overexpression of genes involved in drug resistance including the ABC transporter *MRPA* and several genes related to thiol metabolism. The *MRPA* overexpression was validated by quantitative real-time RT-PCR and further analysis revealed that this increased expression was correlated to gene amplification as part of extrachromosomal linear amplicons in some mutants and as part of supernumerary chromosomes in other mutants. The expression of several other genes encoding hypothetical proteins but also nucleobase and glucose transporter encoding genes were found to be modulated.

**Conclusions/Significance:**

Mechanisms classically found in Old World antimony resistant *Leishmania* were also highlighted in New World antimony-resistant *L. amazonensis*. These studies were useful to the identification of resistance molecular markers.

## Author Summary

*Leishmania* are unicellular microorganisms that can be transmitted to humans by the bite of sandflies. They cause a spectrum of diseases called leishmaniasis, which are classified as neglected tropical diseases by the World Health Organization. The treatment of leishmaniasis is based on the administration of antimony-containing drugs. These drugs have been used since 1947 and still constitute the mainstay for leishmaniasis treatment in several countries. One of the problems with these compounds is the emergence of resistance. Our work seeks to understand how these parasites become resistant to the drug. We studied antimony-resistant *Leishmania amazonensis* mutants. We analyzed gene expression at the whole genome level in antimony-resistant parasites and identified mechanisms used by *Leishmania* for resistance. This work could help us in developing new strategies for treatment in endemic countries where people are unresponsive to antimony-based chemotherapy. The identification of common mechanisms among different species of resistant parasites may also contribute to the development of diagnostic kits to identify and monitor the spread of resistance.

## Introduction

Leishmaniasis refers to a spectrum of parasitic diseases caused by protozoan parasites belonging to the genus *Leishmania*. The diseases are classified as neglected tropical diseases according to the World Health Organization (WHO) and constitute a public health problem in many developing countries of East Africa, the Indian subcontinent and Latin America. Human leishmaniasis has a prevalence of 12 million cases, with an estimated population of 350 million at risk and an incidence of 2 million new cases annually. Depending on *Leishmania* species, the host immune response, and environmental factors, leishmaniasis exhibits a broad spectrum of clinical manisfestations [1]. For example, in the New World, *Leishmania* (*Leishmania*) *amazonensis, Leishmania* (*Viannia*) *guyanensis* and *Leishmania* (*Viannia*) *braziliensis* are the causative agents of cutaneous and mucocutaneous leishmaniasis while *Leishmania* (*L.*) *infantum chagasi* is the aetiological agent of American visceral leishmaniasis [1], [2].

Pentavalent antimonials (Sb^V^), such as sodium stibogluconate (Pentostam®) and meglumine antimoniate (Glucantime®) have been the first-line drugs in the treatment of all forms of leishmaniasis in South America, North Africa, Turkey, Bangladesh and Nepal. One major drawback of the Sb^V^ treatment is the emergence of resistance. For example, more than 60% of patients with visceral leishmaniasis in Bihar State in India are unresponsive to treatment with Sb^V^ antimonials [3]. The emergence of antimony resistance is related to inappropriate drug exposure resulting in a build-up of subtherapeutic blood levels and increasing tolerance of parasites to Sb^V^ [4]. Other drugs have been introduced as alternative chemotherapeutic agents including pentamidine, paromomycin, liposomal amphotericin B and miltefosine. However, either side effects, lower effectiveness or high cost have limited their use [5].

The mechanisms involved in antimony resistance in *Leishmania* are partially understood. Antimonial drugs are administered as Sb^V^, a prodrug that is reduced to Sb^III^, the trivalent and biologically active Sb form [6], [7]. However, the site of this reduction (macrophages and/or parasites) remains unclear. Two genes that encode proteins involved in Sb reduction have been described recently, the arsenate reductase *LmACR2* and *TDR1* thiol-dependent reductase [8], [9]. Nevertheless, the role of these reductases in antimony resistance is not clear. Non enzymatic Sb reduction is also possible and probably mediated by the reducing agents glutathione (GSH) and trypanothione (T(SH)_2_) [5], [10], [11]. Once reduced in the macrophages, Sb^III^ uptake is mediated by the aquaglyceroporin1 (AQP1) [12] and downregulation of *AQP1* gene expression is correlated to resistance [13].

Increases of T(SH)_2_ levels have been observed in parasites selected for resistance to Sb^III^ or arsenite [14]. This enhancement is usually related to the increased levels of rate-limiting enzymes involved in the synthesis of GSH (gamma glutamylcysteine synthetase- γ-GCS) and polyamines (ornithine decarboxylase – ODC) [15], [16]. The use of specific inhibitors of γ-GCS or ODC can revert the resistance phenotype in mutants [16]. The ATP-binding cassette (ABC) protein MRPA has been classically related with drug resistance in *Leishmania* and plays a major role in metal resistance in these parasites [17]. MRPA is a member of the multidrug-resistance protein (MRP) family and its localization in intracellular vesicle membranes strongly suggests that it sequesters Sb-thiol complexes into these vesicles [18]. The *MRPA* gene has been found frequently amplified in laboratory-selected antimony- or arsenite-resistant *Leishmania* mutants as well as in field isolates [19], [20], [21].

Improved knowledge of the mechanisms involved in drug resistance using laboratory-selected mutants or field isolates are mostly derived from Old World *Leishmania* species such as *L. tarentolae* [22], *L. major* [23], [24], *L. tropica* [25], *L. donovani* [26], and *L. infantum* [27]. On the other hand, the mechanism of drug resistance in New World *Leishmania* species remains poorly explored. Nevertheless, phenotypic and molecular characterizations of drug resistance have been recently published for human pathogenic neotropical *Leishmania* species [28], [29], [30]. Resistance to antimony in *L. amazonensis* has not been well studied as yet. Understanding the mechanisms responsible for drug resistance in *Leishmania* could support the design of new strategies for the successful treatment of leishmaniasis as well as the identification of molecular markers for resistance.

Considering the multiplicity of mechanisms leading to antimony resistance, the simultaneous analysis of gene expression could provide useful information about the antimony-resistance mechanisms in *Leishmania* and help the identification of new pathways involved in resistance. Recent studies have demonstrated the usefulness of whole-genome DNA microarrays for studying drug resistance in *Leishmania* [31], [32]. In this study, populations of *L. amazonensis* resistant to Sb^III^ were selected *in vitro* in order to study global gene expression modulation associated with antimony resistance.

## Methods

### Parasite culture conditions and selection of Sb^III^ resistant parasites

*Leishmania amazonensis* (MHOM/BR/1989/Ba199) promastigotes were maintained in minimum essential culture medium (α-MEM) (Gibco, Invitrogen, NY, USA), supplemented with 10% (v/v) heat-inactivated fetal calf serum (Multicell, Wisent Inc. Québec, CA), 100 µg/ml kanamycin, 50 µg/ml ampicillin, 2 mM L-glutamine, 5 µg/ml hemin, 5 µM biopterin, (Sigma-Aldrich, St Louis, USA), pH 7.0 and incubated at 25°C in B.O.D incubators (Johns Scientific-VWR, Toronto, CA). The parasites were kindly provided by Dr. Aldina Barral, Gonçalo Muniz Research Center, Oswaldo Cruz Foundation, Brazil [33]. Populations of *Leishmania amazonensis* promastigotes were selected for Sb^III^ resistance as previously described [19]. The four independent mutants of *L. amazonensis* Ba199Sb^III^2700.1 to Ba199Sb^III^2700.4 were individually selected in 25 cm^2^ flasks containing 5 ml of α-MEM medium in the presence of Sb^III^ concentrations up to 2700 µM.

### Stability and specificity of Sb^III^ resistance *in vitro*

*L. amazonensis* Ba199Sb mutants selected for Sb^III^ resistance were grown in the absence of antimony pressure for 20 passages to test for the resistance stability phenotype [34].

### *Leishmania* full genome microarray design

The full genome arrays were described previously [31], [32], [35]. GeneDB version 3.0 of *L. infantum* genome and *L. major* genome version 5.2 were used for the probe selection. The microarray chip includes a total of 9173 *Leishmania* specific probes and control probes and made by Agilent Technologies (Mississauga, ON, CA). These arrays have been used successfully with several species [31], [35].

### RNA extraction and cDNA labeling

Total RNA was extracted from 10^8^ promastigotes during the mid-log growth phase using RNeasy Plus mini kit (Qiagen Sciences, Maryland, USA) as described by the manufacturer. The quality (based on the appearance of the spectra) and quantity of RNA were assessed using RNA 6000 Nano Assay chips on Bioanalyzer 2100 (Agilent Technologies Santa Clara, CA, USA). For each probe, 7 µg of RNA were converted to aminoallyl-dUTP incorporated cDNA using random hexamers (Roche, Basel, Switzerland) in presence of Superscript III RNase H reverse transcriptase (Invitrogen, Carlsbad, CA, USA). Aminoallyl-dUTP incorporated cDNA were thereafter coupled to Alexa Fluor 555 or Alexa Fluor 647 (Invitrogen, Carlsbad, CA, USA) according to manufacturer recommendations. Fluorescent cDNA were then purified using the probe purification kit ArrayIt (TeleChem International, Sunnyvale, CA, USA) and quantified spectrophotometrically.

### Microarray hybridization

The labeled and purified cDNA from *L. amazonensis* was mixed with 200 µg/ml sonicated salmon sperm DNA (Agilent Technologies, Santa Clara, CA, USA); 200 µg/ml yeast tRNA (Sigma-Aldrich Ltd, ON, CA); 1 x blocking agent buffer (Agilent Technologies, Santa Clara, CA, USA) and 1 x hybridization buffer (Agilent Technologies, Santa Clara, CA, USA), then mixed, denaturated 3 min at 95°C and incubated 30 min at 37°C. Mixed labeled cDNAs were applied in the hybridization chamber (Agilent Technologies, Santa Clara, CA USA) and the hybridization was performed for 24 h at 65°C into a hybridization oven (GeneChip®, Stovall Life Sciences, Greensboro, NC, USA). Slides were washed 5 min at room temperature in 0,5X SSC, 5% Triton-X102 with gentle agitation and subsequently washed 5 min in pre-warmed 0,1X SSC, 0,005% Triton-X102 at room temperature with occasional stirring.

### Microarray data acquisition and analysis

Detection of Alexa Fluor 555 and Alexa Fluor 647 signals were performed on a G2565CA microarray scanner (Agilent Technologies, Santa Clara, CA, USA) at 5 µm resolution as previously described [32]. The signal intensity data were extracted from the primary scanned images using GenePix Pro 6.0 software (Axon Instruments, Union City, CA, USA). Five different cDNA preparations of each Ba199Sb mutant and their respective Ba199 wild-type were analyzed including dye-swaps. Normalization and statistical analyses were performed in R 2.2.1 software using the LIMMA (Linear Models for Microarray Data) 2.7.3 package [36], [37], [38]. Background correction was performed using the “edwards” method; within-array normalization was done by loess and between array normalization by the Aquantile method. Multiple testing corrections were done using the false discovery rate method with a threshold *p* value of 0.05. Only genes statistically significant with an absolute ratio greater than 1.5 were considered. Custom R programs were used for the generation of the chromosome expression maps. Data are available with the GEO accession number GSE26159.

### Quantitative real-time RT-PCR

Three independent RNA preparations were used for each real-time RTPCR experiment. First-strand cDNA was synthesized from 2.5 µg of RNA using Oligo dT_12–18_ and SuperScript II RNase H-Reverse Transcriptase (Invitrogen, Carlsbad, CA, USA) according to the manufacturer protocol. Equal amounts of cDNA were run in triplicate and amplified in 20 µl reactions containing 1 x SYBR® Green Supermix (Bio-Rad, Hercules, CA, USA), 100 nM forward and reverse primers and 1 µl cDNA target. Reactions were carried out using a rotator thermocycler Rotor Gene (RG 3000, Corbett Research, San Francisco, USA). Initially, mixtures were incubated at 95°C for 5 min and then cycled 30 times at 95, 60 and 72°C for 15 sec. No-template controls were used as recommended. Three technical and biological replicates were established for each reaction. The relative amount of PCR products generated from each primer set was determined based on the threshold cycle (Ct) value and the amplification efficiencies. Gene expression levels were normalized to constitutively expressed mRNA encoding glyceraldehyde-3-phosphate dehydrogenase (*GAPDH, LmjF30.2970*). Primers for targeted genes *MRPA* (*LmjF23.0250*), *NT3* (*LmjF13.1210*) and *LmjF26.2680* were designed using Primer Quest^SM^ (www.idtdna.com/Scitools/Applications/Primerquest). The sequences of the primers for *MRPA* are forward 5′-TGAGACACGCCGCATCAAGAGTAT-3′ and reverse 5′-TCAATGCTTCCTGCAGTACGAGGT-3′; for *NT3* are forward 5′-AAGTTCATCTGGCCTCTCATGGCT-3′ and reverse 5′-GATGGTTGCAAACCACTTGTCCGT-3′; for *LmjF26.2680* are forward 5′-ACCCAGTCATTCGTCATGCACTCT-3′ and reverse 5′- ATCTGGTTGACAGCGTCGCAAATG-3′ *;* and for the GAPDH control forward 5′-GAAGTACACGGTGGAGGCTG-3′ and reverse 5′-CGCTGATCACGACCTTCTTC-3′.

### DNA manipulations

Genomic DNA was isolated from *L. amazonensis* Ba199 WT and Ba199 antimony-resistant mutants using DNazol (Invitrogen, Carlsbad, CA, USA) following the manufacturer's instructions. Southern-blots and pulse field gel electrophoresis (PFGE) conditions were done following standard protocols [20]. Genomic DNAs were digested with *Pvu*I and electrophoresed in 1% agarose gel. The fragments were transferred to Hybond^TM^-N+ membrane (Amersham Pharmacia Biotech, Sunnyvale, CA, USA) and submitted to Southern-blot analysis. Chromosomes of *L. amazonensis* Ba199 WT and antimony-resistant mutants were separated by PFGE in which low melting agarose blocks, containing embedded cells (10^8^ log phase cells/ml) were electrophoresed in a contour clamped homogenous electric field apparatus (CHEF Mapper, Bio-Rad, Hercules, CA, USA) in 0,5 x Tris-Borate-EDTA, with buffer circulation at a constant temperature of 14°C and run time of 30 h. *Saccharomyces cerevisiae* chromosomes were used as size markers. DNA was transferred to nylon membranes, cross-linked to the membrane with UV light. The blots were hybridized with [α-^32^P]dCTP labeled DNA probes. The probes used in the present study included a 450 bp *MRPA* fragment and a *α-tubulin* probe used to control the DNA loading.

### Measurement of intracellular thiols

Intracellular thiols were analyzed by derivatizing with mono-bromobimane and separating by high-performance liquid chromatography as described previously [14], [39] using a chromatograph Shimadzu SCL 10A. Thiols were identified from bimane fluorescence with excitation and emission at 360 and 450 nm, respectively using a coupled fluorescence detector (Shimadzu RF-10Axl).

### Statistical analyses

The IC_50_ values were calculated by linear regression using the software GraphPad Prism 5.0 and Sigma Plot 10.0 for windows. Differences in the level of intracellular thiols were analyzed by one-way ANOVA followed by Dunnett's multiple comparison test post-test using GraphPad Prism 5.0. The level of significance acceptable was 95% (*p*<0.05).

## Results

### Characterization of resistance phenotype in laboratory-selected antimony resistant mutants

Four independent mutants of *L. amazonensis* were selected step by step for antimony (Sb^III^) resistance. The IC_50_ value of the sensitive Ba199 strain was 83 µM, whereas the antimony resistant-mutants Ba199Sb^III^2700.1, 2700.2, 2700.3 and 2700.4 had IC_50_ values greater than 2700 µM (Table 1), the highest achievable soluble Sb^III^ concentration in α-MEM medium at pH 7. The stability of the resistance phenotype was tested by growing the cells in the absence of Sb^III^. After 20 passages without drug pressure, only the resistance in mutant Ba199Sb^III^2700.2 was found to be stable, while the other three mutants showed decreased resistance levels (Table 1). However, reversion was only partial since the Ba199Sb^III^2700.1, 2700.3 and 2700.4 mutants were not as sensitive as wild-type cells to Sb^III^ (Table 1). The susceptibility to miltefosine in the Sb^III^-resistant *L. amazonensis* mutants was also tested. None were cross-resistant but 3 out of the 4 lines were surprisingly hypersensitive to it (Table 1). Intriguingly, we have also observed hypersensitivity to miltefosine in *L. infantum* Sb^III^-resistant mutants (W. Moreira and M. Ouellette, unpublished observations).

**Table 1:** Inhibitory concentration of *L. amazonensis* strain Ba199 antimony-sensitive and –resistant independent mutants.

Strain	IC_50_ (µM)		
	Sb^III^	Miltefosine	Sb^III^ (rev)
*Leishmania amazonensis*Ba199 WT	83	7.23	75
Ba199Sb^III^2700.1	>2700>2700>2700>2700	1.37	407
Ba199Sb^III^2700.2		1.57	>2700
Ba199Sb^III^2700.3		9.42	915
Ba199Sb^III^2700.4		3.25	488

The IC_50_ values were also calculated for revertant parasites (rev), which were submitted to 20 passages in absence of Sb^III^. Average of at least 3 measurements with less than 5% variation.

### RNA expression profiling in *Leishmania amazonensis* antimony-resistant mutants

**Figure 1:**
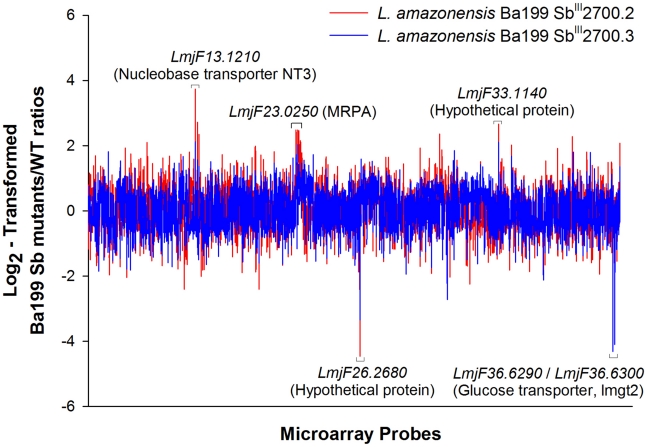
Differential gene expression in *L. amazonensis* antimony resistant mutants. Overlap of log_2_-transformed *L. amazonensis* Ba199 Sb^III^2700.2/WT and Sb^III^2700.3/WT expression ratio plotted as a function of the chromosomal location of probes represented on the full-genome microarrays from chromosome 1 (left end) to chromosome 36 (right end). Vertical lines represent the log_2_-transformed expression ratio of individual genes. Upregulated genes are represented by positive values whereas negative values indicate downregulated genes at the RNA level. The plot represents the average values of five independent hybridizations for each mutant.

**Figure 2:**
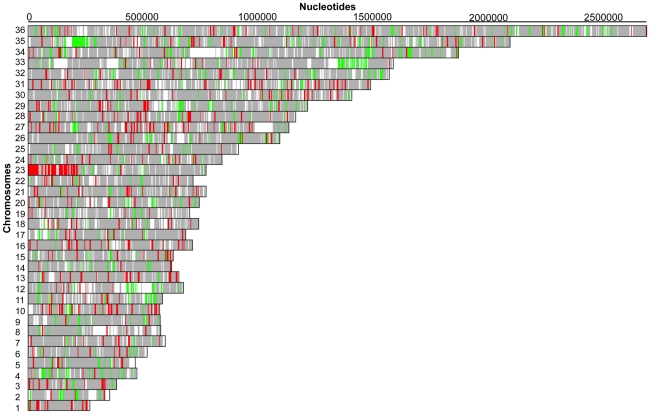
Gene expression map of *L. amazonensis* Ba199 Sb^III^2700.2/WT. DNA microarrays data were analyzed by custom R programs to illustrate the expression profile of Ba199Sb^III^2700.2/WT by extrapolating on a chromosome map of *L. major.* Red lines indicate upregulated genes in Ba199 Sb^III^2700.2, whereas green lines indicate downregulated genes. Gray features indicate genes equally expressed in both samples while white regions have not hybridized to probes.

**Figure 3:**
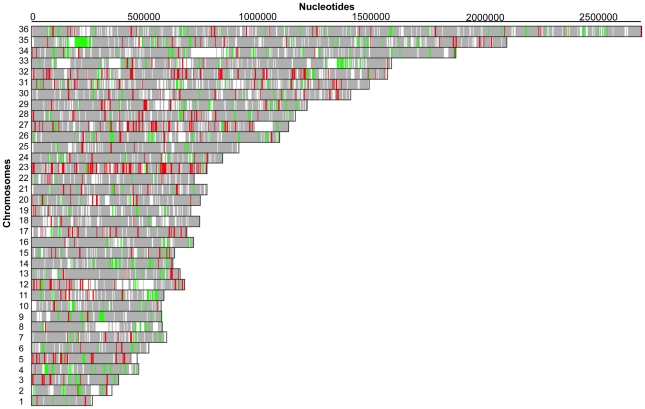
Gene expression map of *L. amazonensis* Ba199 Sb^III^2700.3/WT. DNA microarrays data were analyzed by custom R programs to illustrate the expression profile of Ba199Sb^III^2700.3/WT by extrapolating on a chromosome map of *L. major.* Red lines indicate upregulated genes in Ba199 Sb^III^2700.3, whereas green lines indicate downregulated genes. Gray features indicate genes equally expressed in both samples while white regions have not hybridized to probes.

The Ba199Sb^III^2700.2 and Ba199Sb^III^2700.3 lines were selected for gene expression studies using full genome DNA microarrays. We plotted the log_2_-transformed gene expression ratios of Ba199Sb^III^2700.2 (red line) and Ba199Sb^III^2700.3 (blue line) compared to Ba199WT parental strain, as a function of the microarray probes (Fig. 1). Most genes were equally expressed but about 10% of genes showed a statistical significant variation (summarized in Table S1 and detailed in Tables S2 and S3) with approximately 2-fold differential expression but some reached log_2_-transformed ratio values up to 4 and −4 (Fig. 1). The differential hybridization data were also represented on a chromosome by chromosome basis (Figs. 2 and 3). Upregulated and downregulated genes are indicated by red and green lines, respectively, while equally expressed genes were shown as gray regions. Some obvious changes in gene expression were noticed. A specific region at one telomeric end of chromosome 23 was upregulated in Ba199Sb^III^2700.2 (Fig. 2), while most genes of chromosome 23 seemed upregulated in Ba199Sb^III^2700.3 (Fig. 3). Chromosome aneuploidy has been described previously in Old World drug resistant *Leishmania* [31], [32] and the chromosome maps of Figs. 2 and 3 suggest that this phenomenon also takes place in New World *Leishmania* species with chromosomes 1, 10, 16, 27 and 31 becoming polyploids in Ba199Sb^III^2700.2 (Fig. 2) while in addition to chromosome 23, chromosomes 5, 27 and 32 are polyploids and chromosome 4 is haploid in Ba199Sb^III^2700.3 (Fig. 3). A region of chromosome 35, 250 kb from one telomeric end, corresponds to loci where the expression of genes was down regulated in both Ba199Sb^III^2700.2 and Ba199Sb^III^2700.3 mutants (Figs. 2 and 3). The expression of genes part of a region on chromosome 33, 1.5 Mb from one telomere end was also down regulated in both mutants (Figs. 2 and 3).

**Figure 4:**
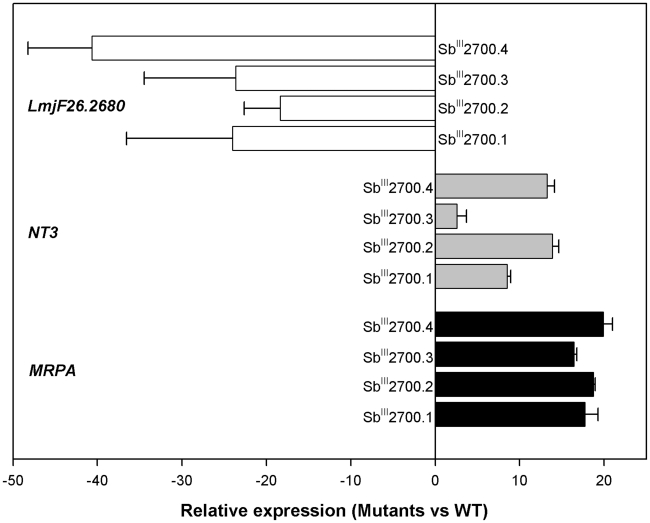
RNA expression in *L. amazonensis* antimony resistant mutants by quantitative real time RT-PCR. The RNA expression ratios of the nucleobase transporter gene *NT3*, the ABC protein *MRPA* gene and the hypothetical protein encoding gene *LmjF26.2680* were measured in *L. amazonensis* antimony resistant mutants, which were compared to levels found in WT cells. The expression of *GAPDH* was used to normalize the data. The values are the mean of two independent experiments each performed with three biological RNA preparations.

**Figure 5:**
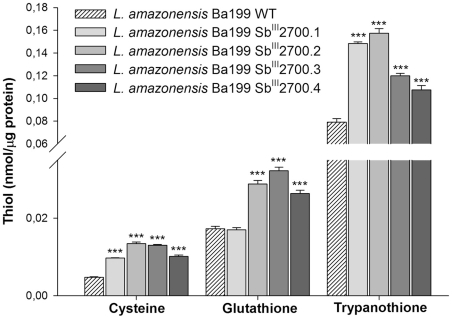
Intracellular levels of thiols in *L. amazonensis* antimony resistant mutants. Thiols were derivatized with monobromobimane and separated by HPLC coupled with a fluorescent detector. Values are representative of two experiments performed in triplicate. Asterisks indicate statistically significant changes compared with Ba199 WT. The data were analyzed by one-way ANOVA followed by Dunnett's multiple comparison test. ***  =  *p<*0.01.

The array results led to several candidate genes putatively correlated to resistance. Candidate genes could either be highly differentially regulated or part of large regions differentially regulated, as highlighted in Figs. 2 and 3. The genes common to both mutants most differentially down regulated included the hypothetical protein gene *LmjF26.2680* and a putative lmgt2 glucose transporter gene *LmjF36.6290* (Fig. 1 and Supplementary Tables S2 and S3). On the other hand, the gene common to both mutants most upregulated was corresponding to the nucleobase transporter NT3 *LmjF13.1210*. The overexpression of *NT3* was confirmed by qRT-RTPCR which yielded similar results as found with microarrays with higher expression of *NT3* in Ba199Sb^III^2700.2 compared to 2700.3 (Fig. 1, Fig. 4). We also tested the two other *L. amazonensis* mutants available and found that *NT3* was also overexpressed in Ba199Sb^III^2700.1 and 2700.4 (Fig. 4). Similarly, we confirmed the down regulation of *LmjF26.2680* by qRT-RTPCR not only in Ba199Sb^III^2700.2 and 2700.3 but also in two other *L. amazonensis* resistant mutants (Fig. 4). None of the genes described above were previously linked to antimony resistance in *Leishmania*. For specific larger regions that were presumed to be up or down regulated as determined from the chromosome maps of Figs. 2 and 3, we found that the region of chromosome 23 upregulated in Ba199Sb^III^2700.2 (Fig. 2) contained several genes (Table S2) including the ABC protein gene MRPA *LmjF23.0250*, a well established marker of antimony resistance [40], [41]. The *MRPA* gene was also upregulated in Ba199Sb^III^2700.3 (Fig. 3) as determined by microarrays (Table S3). As discussed above, two regions of chromosome 35 and 33 appeared to be down regulated in both mutants. The region of chromosome 35 encodes for several hypothetical proteins, but also three proteophosphoglycan (*PPG*) genes *PPG1*, *PPG3* and *PPG5* (Tables S2 and S3). Similarly, the region of chromosome 33 corresponds mostly to hypothetical proteins (Tables S2 and S3). With the exception of *MRPA*, none of the genes highlighted in this study were previously linked to antimony resistance. We searched for genes that were previously linked to resistance with significant changes in gene expression and found several genes that were upregulated in the Ba199Sb^III^ mutants and that were involved in redox and thiol metabolism such as peroxidoxin (*LmjF23.0040*), glutaredoxin (*LmjF05.0310*), trypanothione synthetase (*LmjF23.0460*; *LmjF27.1870*), trypanothione reductase (*LmjF05.0350*), and spermidine synthase (*LmjF04.0580*) (Tables S2 and S3). The overexpression of several trypanothione biosynthetic genes (e.g. spermidine synthase, trypanothione synthetase) prompted us to quantify the level of intracellular reduced thiols, since resistance to Sb^III^ is often correlated to increased glutathione and trypanothione levels in Old World *Leishmania* [42]. The antimony-resistant *L. amazonensis* mutants, with the exception of Ba199Sb^III^2700.1 (for glutathione), had significant higher levels of cysteine, glutathione and trypanothione (Fig. 5).

We also tested the role of genes previously not associated with resistance, concentrating on some of the genes most differentially expressed. These genes correspond to the hypothetical gene *LmjF26.2680* which was down regulated by more than 20-fold in all mutants (Fig. 4) and NT3 that was overexpressed in all mutants as determined by real-time RT-PCR (Fig. 4). Transfection of *LmjF26.2680* in wild-type *L amazonensis* or in its resistant mutants did not change their susceptibilities to Sb^III^ (results not shown). Similarly, transformation and overexpression of *NT3*, did not lead to higher resistance to Sb^III^ in wild type cells (result not shown).

### Increased MRPA expression mediated by gene amplification in antimony-resistant *L. amazonensis* mutants

**Figure 6:**
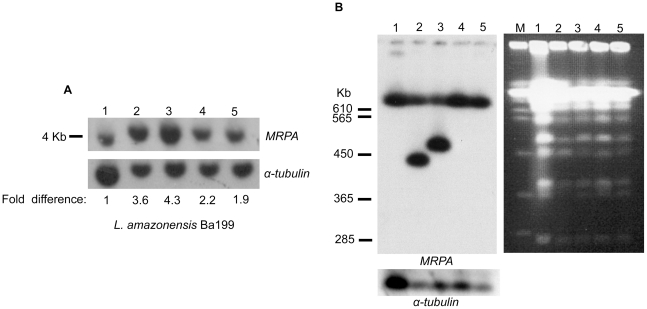
Amplification of *MRPA* gene in antimony-resistant *L. amazonensis.* (**A**) Total genomic DNAs of *Leishmania* cells were digested with *PvuI*, electrophoresed on agarose gel, transferred onto a membrane and hybridized with an *MRPA* specific probe. Southern blot hybridization was quantified using ImageJ 1.43 (NIH) and the fold differences in DNA copy number of Ba199Sb mutants compared to WT are listed. (**B**) Pulsed field gel electrophoresis (PFGE) was used to separate the chromosomes of *L. amazonensis*. The CHEF blot was hybridized with the same *MRPA* probe. The Southern-blot membranes were washed and rehybridized with an α-tubulin probe to monitor the amount of DNA layered on the gel. Marker (M); *L. amazonensis* Ba199WT (lane 1); Ba199Sb^III^2700.1 (lane 2); Ba199Sb^III^2700.2 (lane 3); Ba199Sb^III^2700.3 (lane 4); Ba199Sb^III^2700.4 (lane 5).

The *MRPA* gene was overexpressed in both mutants (Table S2 and S3) and this upregulation was indeed confirmed by qRT-RTPCR in Ba199Sb^III^2700.2 and 2700.3 but *MRPA* was also found overexpressed in Ba199Sb^III^2700.1 and 2700.4 (Fig. 4). The fold increased expression by qRT-RTPCR was higher than what microarray would have suggested. Often, but not always, gene overexpression is correlated to gene amplification in *Leishmania* [13], [40], [41]. Southern blot analysis and careful densitometric quantification has indeed indicated that *MRPA* gene copy number is increased in the mutants compared to wild-type cells (Fig. 6A). Increased gene copy number is usually due to the formation of extrachromosomal circular or linear elements [43], [44] although changes in copy number of whole chromosomes have also been reported [31], [32]. Search for extrachromosomal circles failed by standard alkaline lysis extractions and we thus relied on CHEF gels to separate the *Leishmania* chromosomes and investigated for the presence of short linear amplicons. Hybridization to a *MRPA* probe showed the presence of linear amplicons in Ba199Sb^III^2700.1 and 2700.2 while the whole chromosome 23 was increased in copy number in Ba199Sb^III^2700.3 and 2700.4 (Fig. 6B). These results are consistent with the microarray data (Figs. 2 and 3).

## Discussion

Resistance to antimony in *Leishmania* has been studied mostly in Old World species and mostly in strains in which resistance was induced under laboratory conditions (reviewed in [5], [42]). However, with a better understanding of *in vitro* resistance mechanisms, more work has recently been done with clinical isolates and some of the markers highlighted in *in vitro* studies were shown to correlate with drug resistance in clinical isolates [20], [45]. In general, there is a reasonable agreement between *in vitro* susceptibility testing and clinical response with Old World *Leishmania* when assays are carried out with intracellular parasites [45], [46], [47]. However, there are conflicting results in linking *in vitro* susceptibility testing and clinical responses with New World leishmaniasis [48], [49]. There have been few studies on mechanisms of resistance to antimony in New World parasites and we have thus used here the proven approach of *in vitro* selected resistant cells. Four independent *L. amazonensis* clones were selected for resistance to Sb^III^. Resistance was in general unstable when cells were grown in absence of the drug (Table 1), a result also recently observed with New World *Leishmania* selected for antimony resistance [28].

To find possible markers of resistance in these *L. amazonensis* strains, we carried out RNA expression profiling on full genomic DNA microarrays, a technique proven useful to study resistance mechanisms in *Leishmania* [31], [32], [50]. We found several gene candidates (Table S2 and S3), some for which the expression was highly modulated in comparison to sensitive isolates. Two of these genes (the hypothetical *LmjF26.2680* and *NT3*) were new and were experimentally tested by gene transfection. However, we could not directly link them to resistance. *NT3* and LmjF26.2680 were respectively overexpressed and down-regulated in four independent mutants (Fig. 4), and this recurrence would argue for some role in resistance. If it is not directly involved in resistance as the transfection work would suggest, it could either require another product to confer resistance or it may have another more indirect role such as in increased fitness or compensating for other mutations. We noticed that one glucose transporter in Ba199Sb^III^2700.3 was down regulated (Fig. 1). Decrease glucose uptake, for example by minimizing reactive oxygen species, was suggested as a general mechanism associated with drug resistance in *L. amazonensis* [51]. Future work will be required to test this. It is also worthnoting that while the expression of *NT3* is increased, this is not due to gene amplification. Indeed, the *NT3* copy number remains similar to wild-type (result not shown). While changes in expression in resistant isolates are often due to changes in gene copy number, there has been several other reports of increased expression by other means which will likely involve post-transcriptional regulation mechanisms. Indeed, the expression of genes in *Leishmania* is not controlled at the level of transcription initiation [52], [53].

The microarray work allowed detecting alterations of expression of large regions of genomic DNA and even of whole chromosomes (Figs. 2 and 3). In *Leishmania* these alterations are usually linked to changes in copy number [31], [32]. One region that attracted our attention was part of chromosome 23. Mutant Ba199Sb^III^2700.2 had a specific region that was overexpressed while the whole chromosome 23 seemed overexpressed in Ba199Sb^III^2700.3. The gene *MRPA*, one marker highly correlated to Sb^III^ resistance in Old World *Leishmania*, is encoded by chromosome 23. We tested whether this increased expression was due to changes in copy number and Southern blot analysis indeed confirmed that *MRPA* is amplified (Fig.6A). New World *Leishmania* is divided in two subgenus: *Leishmania* and *Viannia*. Gene amplification is rare in the *Viannia* subgenus [30] and this may be due to an active RNA interference (RNAi) mechanism in this subgenus but absent in the *Leishmania* subgenus [54]. It is thus surprising that there is one report of a circular extrachromosomal amplification of *MRPA* in *L. V. guyanensis* selected for antimony resistance [55]. There is, however, ample report of gene amplification in the New World *Leishmania* subgenus whether it is *L. amazonensis* [56], [57] or *L. mexicana* [58]. No *MRPA* amplification has been observed in one *L. amazonensis* strain selected for Sb^III^ resistance [59] but a circular amplification was observed in *L. mexicana* selected for resistance to the related metal arsenite [58]. The *MRPA* containing amplicon in *L. mexicana* or *L. V. guyanensis* corresponded to an extrachromosomal circle. In Ba199Sb^III^2700.2 the amplification was a linear amplicon and extended from the telomeric region to gene *LmjF23.0540* (a region of ∼230 kb). All linear amplifications so far described, indeed extended to the telomeric region and are usually forming large inverted duplications [31]. This duplication of the region amplified fits with the size of this linear amplicon (Fig. 6B). Interestingly, we also found an *MRPA* containing linear amplicon in Ba199Sb^III^2700.1 (Fig. 6B). The amplicon is smaller, suggesting that a different rearrangement point, usually at the level of inverted repeats [31], [60] has been used. The microarray data indicate that the whole chromosome 23 was increased in Ba199Sb^III^2700.3 and this was corroborated by Southern blot analysis (Fig. 6). Indeed, the CHEF showed clearly that chromosome 23 had a higher hybridization intensity compared to Ba199Sb^II^2700.2 (Fig. 6B). Interestingly, polyploidy of chromosome 23 was also observed in Ba199Sb^II^2700.4. Intriguingly, this relatively modest increase in copy number was nonetheless correlated to a high MRPA expression at the RNA level (Fig. 4). Thus an increase in MRPA expression in *L. amazonensis* is correlated to either the formation of extrachromosomal linear amplicons or the increased ploidy of the chromosome.

This study has shown that mechanisms of resistance to antimony found in Old World *Leishmania* can also be detected in New World species. This includes higher thiol levels (Fig. 5) and increased expression of the ABC MRPA, whose gene product sequesters thiol-metal conjugates into an intracellular organelle [18]. Overexpression of several genes was found to correlate with increased thiols [15], [16], [50] and overexpression of spermidine synthase (leading to polyamines, one constituent of trypanothione) and trypanothione synthase (supplementary Tables S2 and S3) could contribute to the observed increased thiols. Also we noticed that trypanothione reductase was overexpressed and this would maintain thiols into a reduced form and this gene was found overexpressed in field isolates [61]. Many other genes were found to be differentially regulated although analysis of two candidates did not allow finding a role in resistance. Nonetheless with all microarray experiments done with several different species it should now be possible to perform meta-analysis which could direct at further candidates for a better understanding of antimony resistance mechanisms in the protozoan parasite *Leishmania*.

The study presented here should serve as a useful basis for analyzing antimony resistance in clinical isolates of new world leishmaniasis. Indeed, *in vitro* work mostly with the promastigote stage of old world leishmaniasis has led to a number of drug resistant markers [5], [42]. These markers were shown to confer resistance in the amastigote or intracellular stage of the parasite [40] and even more importantly in *L. donovani* field isolates [20], [61], [62], [63]. Since several markers were highlighted here with *in vitro* resistance in *L. amazonensis,* it would now be possible to test whether similar resistance mechanisms take place with drug resistant clinical isolates of New World leishmaniasis.

## Supporting Information

Table S1 Overview of differential gene expression profile in laboratory-selected antimony-resistant mutants *Leishmania amazonensis* Ba199Sb^III^2700.2 and Ba199Sb^III^2700.3
(DOC)

Table S2 Genes significantly modulated in antimony-resistant *Leishmania amazonensis* Ba199Sb^III^2700.2. The data were obtained by full genome microarray hybridization of Ba199Sb^III^2700.2 against Ba199 WT.
(DOC)

Table S3 Genes significantly modulated in antimony-resistant *Leishmania amazonensis* Ba199Sb^III^2700.3. The data were obtained by full genome microarray hybridization of Ba199Sb^III^2700.3 against Ba199 WT.
(DOC)
