# Supplementary material for: Gene Expression Profiling and Molecular Characterization of Antimony Resistance in Leishmania amazonensis
Source: PLoS Negl Trop Dis. 2011 May 24;5(5):e1167. doi: 10.1371/journal.pntd.0001167 (PMC3101167; doi:10.1371/journal.pntd.0001167)
Supplement: Table S1 — Overview of differential gene expression profile in laboratory-selected antimony-resistant mutants Leishmania amazonensis Ba199SbIII2700.2 and Ba199SbIII2700.3 (DOC) [file pntd.0001167.s001.doc]

**Table S1** – Overview of differential gene expression profiles in laboratory-selected antimony-resistant mutants *Leishmania amazonensis* Ba199Sb^III^2700.2 and Ba199Sb^III^2700.3^a^

|  | ***L. amazonensis* modulated genes** | | | |
| --- | --- | --- | --- | --- |
| **Differential expression (fold)** | **Ba199Sb^III^2700.2** | | **Ba199Sb^III^2700.3** | |
|  | Upregulated | Downregulated | Upregulated | Downregulated |
| 1.68-1.99 | 278 | 204 | 255 | 197 |
| 2-2.99 | 230 | 221 | 168 | 157 |
| ≥ 3 | 51 | 50 | 17 | 21 |
| **Total** | 559 | 475 | 440 | 375 |

^a^ A total of 1849 genes presented here showed at least 1.68 in fold expression and minimum level of significance at 95% (*p* value *<* 0.05).
