# Supplementary material for: Gene Expression Profiling and Molecular Characterization of Antimony Resistance in Leishmania amazonensis
Source: PLoS Negl Trop Dis. 2011 May 24;5(5):e1167. doi: 10.1371/journal.pntd.0001167 (PMC3101167; doi:10.1371/journal.pntd.0001167)
Supplement: Table S2 — Genes significantly modulated in antimony-resistant Leishmania amazonensis Ba199SbIII2700.2. The data were obtained by full genome microarray hybridization of Ba199SbIII2700.2 against Ba199 WT. (DOC) [file pntd.0001167.s002.doc]

| **Gene Systematic ID** | **GeneDescription** | **Fold Difference** |
| --- | --- | --- |
| **Downregulated genes** | | |
| **LmjF01.0010**  **Table S2.** Genes significantly modulated in antimony-resistant *Leishmania amazonensis* Ba199Sb^III^2700.2. The data were obtained by full genome microarray hybridization of Ba199Sb^III^2700.2 against Ba199WT. | **hypothetical protein, unknown function** | **-2,26** |
| **LmjF01.0260** | **hypothetical protein, conserved** | **-2,84** |
| **LmjF02.0040** | **aminopeptidase P1, putative** | **-1,76** |
| **LmjF02.0300** | **ABC1 transporter, putative** | **-3,21** |
| LmjF02.0310 | hypothetical protein, conserved | -1,92 |
| **LmjF02.0470** | **hypothetical protein, unknown function** | **-2,44** |
| **LmjF02.0590** | **hypothetical protein, conserved** | **-3,60** |
| **LmjF03.0470** | **hypothetical protein, conserved** | **-2,64** |
| **LmjF03.0530** | **hypothetical protein, conserved** | **-2,65** |
| LmjF03.0660 | hypothetical protein, conserved | -2,19 |
| **LmjF03.0900** | **peter pan protein, putative** | **-1,95** |
| LmjF04.0070 | hypothetical protein, conserved | -1,68 |
| **LmjF04.0110** | **hypothetical protein, conserved** | **-2,16** |
| **LmjF04.0170** | **hypothetical protein, conserved in leishmania** | **-2,70** |
| **LmjF04.0180** | **surface antigen-like protein** | **-2,47** |
| **LmjF04.0190** | **surface antigen-like protein** | **-3,62** |
| **LmjF04.0200** | **surface antigen-like protein** | **-3,91** |
| **LmjF04.0210** | **surface antigen-like protein** | **-2,49** |
| LmjF04.0230 | hypothetical protein, conserved | -1,90 |
| **LmjF04.0280** | **adenosine monophosphate deaminase, putative** | **-1,91** |
| LmjF04.0390 | hypothetical protein | -2,07 |
| **LmjF04.0440** | **serine/threonine protein kinase-like protein** | **-2,31** |
| **LmjF04.0450** | **calpain-like cysteine peptidase, putative** | **-1,85** |
| **LmjF04.0640** | **hypothetical protein** | **-3,04** |
| **LmjF04.0690** | **hypothetical protein** | **-1,93** |
| **LmjF04.0800** | **hypothetical protein, conserved** | **-2,28** |
| **LmjF04.0830** | **hypothetical protein, conserved** | **-1,77** |
| LmjF04.0910 | hypothetical protein, conserved | -1,69 |
| **LmjF04.1020** | **hypothetical protein, conserved** | **-2,05** |
| **LmjF04.1030** | **hypothetical protein, conserved** | **-2,45** |
| **LmjF04.1110** | **proton motive ATPase, putative** | **-2,59** |
| **LmjF04.1200** | **hypothetical protein, conserved** | **-1,83** |
| **LmjF04.1230** | **actin** | **-2,58** |
| LmjF05.0070 | dynein light chain, putative | -1,73 |
| LmjF05.0330 | DNA replication licensing factor, putative | -1,84 |
| LmjF05.0340 | hypothetical protein, conserved | -1,76 |
| **LmjF05.0420** | **dynein-light chain-protein, putative** | **-3,30** |
| LmjF05.0540 | hypothetical protein, conserved | -1,68 |
| LmjF05.0840 | hypothetical protein, unknown function | -1,75 |
| LmjF05.0900 | surface antigen-like protein | -3,38 |
| LmjF05.0960 | dipeptidyl-peptidase III, putative | -1,72 |
| **LmjF06.0130** | **hypothetical protein, conserved** | **-4,13** |
| **LmjF06.0150** | **hypothetical protein, conserved** | **-1,78** |
| **LmjF06.0450** | **hypothetical protein, unknown function** | **-2,36** |
| **LmjF06.0750** | **hypothetical protein, conserved** | **-3,49** |
| **LmjF06.1050** | **protein disulfide isomerase** | **-2,30** |
| **LmjF06.1080** | **hypothetical protein, unknown function** | **-1,69** |
| LmjF06.1120 | hypothetical protein, conserved | -2,14 |
| **LmjF06.1150** | **hypothetical protein, conserved** | **-1,97** |
| **LmjF06.1190** | **hypothetical protein, conserved** | **-2,20** |
| **LmjF07.0370** | **hypothetical protein, unknown function** | **-2,32** |
| **LmjF07.0420** | **hypothetical protein, conserved** | **-2,46** |
| **LmjF07.0890** | **hypothetical protein, unknown function** | **-2,60** |
| **LmjF07.1055** | **hypothetical protein, conserved** | **-1,94** |
| **LmjF08.0140** | **DNA repair protein, putative** | **-2,48** |
| LmjF08.0230 | hypothetical protein, conserved | -1,71 |
| **LmjF08.0460** | **hypothetical protein, unknown function** | **-1,82** |
| LmjF08.0540 | hypothetical protein, conserved | -2,26 |
| **LmjF08.0570** | **hypothetical protein, conserved** | **-1,73** |
| **LmjF09.0300** | **hypothetical protein, conserved** | **-2,90** |
| **LmjF09.0420** | **tyrosine phosphatase, putative** | **-2,38** |
| **LmjF09.0530** | **leucine-rich repeat protein, putative** | **-2,39** |
| **LmjF09.0610** | **hypothetical protein, conserved** | **-1,92** |
| LmjF09.0660 | hypothetical protein, conserved | -1,71 |
| LmjF09.0700 | hypothetical protein, conserved | -1,91 |
| LmjF09.1110 | hypothetical protein, conserved | -1,73 |
| LmjF09.1290 | hypothetical protein, conserved | -1,85 |
| LmjF09.1320 | paraflagellar rod component, putative | -1,92 |
| **LmjF09.1520** | **hypothetical protein, conserved** | **-1,84** |
| **LmjF10.0230** | **hypothetical protein, conserved** | **-2,51** |
| **LmjF10.0370** | **pteridine transporter ft4, putative** | **-2,52** |
| **LmjF10.0385** | **pteridine transporter ft3, putative** | **-2,57** |
| **LmjF10.0390** | **pteridine transporter ft3, putative** | **-3,22** |
| LmjF10.0640 | hypothetical protein, conserved | -1,93 |
| **LmjF10.1140** | **hypothetical protein, conserved** | **-2,50** |
| LmjF11.0050 | SNF2/RAD54 related DNA helicase, putative | -1,69 |
| **LmjF11.0160** | **hypothetical protein, conserved** | **-1,81** |
| LmjF11.0200 | hypothetical protein, conserved | -1,79 |
| **LmjF11.0320** | **PIF1 helicase-like protein, putative** | **-2,46** |
| LmjF11.0840 | hypothetical protein, conserved | -1,71 |
| LmjF11.1040 | hypothetical protein, conserved | -2,42 |
| **LmjF11.1240** | **ABC1 transporter, putative** | **-2,05** |
| **LmjF11.1270** | **ABC transporter, putative** | **-2,86** |
| **LmjF11.1290** | **ABC transporter, putative** | **-3,45** |
| **LmjF11.1310** | **hypothetical protein, conserved** | **-2,76** |
| **LmjF12.0150** | **leucine rich repeat protein, putative** | **-2,91** |
| LmjF12.0170 | hypothetical protein, unknown function | -1,72 |
| LmjF12.0460 | hypothetical protein, unknown function | -2,33 |
| LmjF12.0760 | surface antigen protein 2, putative | -2,56 |
| LmjF12.0920 | surface antigen protein | -2,64 |
| LmjF12.0960 | surface antigen protein 2, putative | -3,20 |
| **LmjF12.1015** | **hypothetical protein, unknown function** | **-2,30** |
| **LmjF12.1040** | **surface antigen protein, putative** | **-3,11** |
| **LmjF12.1060** | **surface antigen protein, putative** | **-3,15** |
| **LmjF12.1070** | **surface antigen protein 2, putative** | **-5,32** |
| LmjF12.1090 | surface antigen protein, putative | -1,99 |
| LmjF12.1140 | n-ethylmaleimide reductase-like protein | -2,67 |
| **LmjF12.1290** | **hypothetical protein, conserved** | **-2,78** |
| **LmjF13.0190** | **hypothetical protein, unknown function** | **-1,93** |
| **LmjF13.0390** | **alpha tubulin** | **-2,51** |
| **LmjF13.1530** | **phospholipid-transporting ATPase 1-like protein** | **-2,41** |
| LmjF14.0300 | hypothetical protein, conserved | -4,04 |
| LmjF14.0490 | hypothetical protein, unknown function | -1,76 |
| **LmjF14.0510** | **stearic acid desaturase, putative** | **-3,53** |
| LmjF14.0625 | hypothetical protein, conserved | -1,74 |
| LmjF14.0630 | hypothetical protein, unknown function | -2,23 |
| **LmjF14.0700** | **fatty acid elongase, putative** | **-4,19** |
| **LmjF14.0720** | **fatty acid elongase, putative** | **-3,50** |
| **LmjF14.0870** | **hypothetical protein, conserved** | **-1,71** |
| **LmjF14.0920** | **mitochondrial DNA polymerase I protein C, putative** | **-2,02** |
| **LmjF14.1080** | **hypothetical protein, conserved** | **-3,02** |
| **LmjF14.1220** | **hypothetical protein, conserved** | **-2,02** |
| **LmjF14.1250** | **hypothetical protein, unknown function** | **-2,17** |
| **LmjF14.1390** | **hypothetical protein, conserved** | **-2,69** |
| **LmjF15.0110** | **hypothetical protein, conserved** | **-1,72** |
| **LmjF15.0350** | **hypothetical protein, conserved** | **-2,23** |
| LmjF15.0630 | hypothetical protein, unknown function | -1,95 |
| LmjF15.0790 | hypothetical protein, unknown function | -1,69 |
| **LmjF15.1150** | **developmentally regulated protein, putative** | **-1,68** |
| **LmjF15.1480** | **cAMP specific phosphodiesterase, putative** | **-2,07** |
| **LmjF15.1510** | **hypothetical protein, conserved** | **-2,41** |
| **LmjF16.0150** | **hypothetical protein, conserved** | **-2,09** |
| LmjF16.0340 | hypothetical protein, conserved | -1,84 |
| LmjF16.0970 | hypothetical protein, conserved | -1,99 |
| **LmjF16.1250** | **hypothetical protein, conserved** | **-1,74** |
| LmjF17.0020 | hypothetical protein, conserved | -1,71 |
| **LmjF17.0170** | **hypothetical protein, conserved** | **-2,08** |
| LmjF17.0200 | receptor-type adenylate cyclase a | -1,99 |
| **LmjF17.0237** | **receptor-type adenylate cyclase b** | **-2,66** |
| LmjF17.0610 | hypothetical protein, unknown function | -1,97 |
| LmjF17.0650 | hypothetical protein, conserved | -2,10 |
| LmjF17.0680 | hypothetical protein, conserved | -1,74 |
| LmjF17.0860 | hypothetical protein, unknown function | -2,45 |
| LmjF17.1030 | hypothetical protein, conserved | -2,12 |
| **LmjF18.0180** | **hypothetical protein, unknown function** | **-2,48** |
| **LmjF18.0270** | **protein kinase, putative** | **-2,42** |
| **LmjF18.0470** | **hypothetical protein, conserved** | **-1,73** |
| LmjF18.0640 | serine/threonine kinase-like protein, putative | -2,19 |
| **LmjF18.0760** | **hypothetical protein, conserved** | **-1,75** |
| LmjF18.1070 | vacuolar protein sorting complex subunit, putative | -1,81 |
| **LmjF18.1230** | **hypothetical protein, conserved** | **-2,39** |
| LmjF18.1640 | hypothetical protein, conserved | -2,23 |
| **LmjF19.0430** | **hypothetical protein, conserved** | **-2,05** |
| LmjF19.0520 | hypothetical protein, conserved | -1,84 |
| LmjF19.0780 | hypothetical protein, conserved | -1,76 |
| **LmjF19.0848** | **microtubial binding protein-like protein** | **-3,68** |
| **LmjF19.0850** | **microtubule associated protein-like protein** | **-2,53** |
| **LmjF19.0910** | **microtubule associated protein-like** | **-3,48** |
| **LmjF19.1100** | **hypothetical protein, conserved** | **-2,47** |
| LmjF19.1140 | hypothetical protein, unknown function | -1,83 |
| **LmjF19.1360** | **hypothetical protein, unknown function** | **-5,32** |
| **LmjF20.0150** | **hypothetical protein** | **-2,58** |
| **LmjF20.0310** | **hypothetical protein, conserved** | **-2,12** |
| **LmjF20.0600** | **hypothetical protein, conserved** | **-2,18** |
| LmjF20.1020 | hypothetical protein, unknown function | -2,15 |
| **LmjF20.1040** | **hypothetical protein, conserved** | **-2,41** |
| LmjF20.1185 | calpain-like cysteine peptidase, putative | -2,05 |
| LmjF20.1185 | calpain-like cysteine peptidase, putative | -2,16 |
| LmjF20.1190 | calpain-like cysteine peptidase, putative | -1,78 |
| LmjF20.1220 | hypothetical protein, conserved | -2,15 |
| LmjF20.1400 | axoneme central apparatus protein, putative | -1,74 |
| LmjF20.1470 | hypothetical protein, unknown function | -1,75 |
| LmjF20.1480 | zinc-binding phosphatase, putative | -2,72 |
| LmjF20.1550 | aminoacylase, putative | -1,95 |
| LmjF20.1560 | aminoacylase, putative | -1,71 |
| LmjF20.1560 | aminoacylase, putative | -1,75 |
| LmjF21.0460 | hypothetical protein, unknown function | -1,75 |
| **LmjF21.0875** | **hypothetical protein, conserved** | **-2,30** |
| **LmjF21.0960** | **hypothetical protein, conserved** | **-2,19** |
| **LmjF21.0970** | **hypothetical protein, conserved** | **-2,01** |
| LmjF21.1170 | surface antigen-like protein | -1,90 |
| **LmjF21.1520** | **hypothetical protein, unknown function** | **-3,18** |
| **LmjF22.0040** | **hypothetical protein, conserved** | **-2,08** |
| **LmjF22.0250** | **hypothetical protein, unknown function** | **-1,95** |
| **LmjF22.0450** | **hypothetical protein, conserved** | **-2,65** |
| LmjF22.0540 | hypothetical protein, conserved | -1,78 |
| **LmjF22.1070** | **hypothetical protein, conserved** | **-2,57** |
| LmjF22.1170 | hypothetical protein, conserved | -1,83 |
| **LmjF23.0125** | **cyclophilin type peptidyl-prolyl cis-trans isomerase, putative** | **-2,01** |
| LmjF23.0610 | hypothetical protein, conserved | -1,85 |
| LmjF23.0780 | hypothetical protein, conserved | -2,51 |
| LmjF23.1020 | hypothetical protein, unknown function | -1,79 |
| **LmjF23.1088** | **hydrophilic surface protein 2** | **-2,48** |
| **LmjF23.1550** | **hypothetical protein, conserved** | **-1,91** |
| **LmjF23.1660** | **metallo-beta-lactamase family protein-like protein** | **-2,70** |
| LmjF24.0160 | hypothetical protein, conserved | -2,23 |
| **LmjF24.0280** | **dynein intermediate-chain-like protein** | **-2,07** |
| **LmjF24.0340** | **hypothetical protein, conserved** | **-1,72** |
| **LmjF24.0400** | **hypothetical protein, conserved** | **-2,77** |
| **LmjF24.1160** | **hypothetical protein, unknown function** | **-1,95** |
| **LmjF24.1560** | **hypothetical protein, conserved** | **-2,51** |
| **LmjF24.1600** | **hypothetical protein, unknown function** | **-2,01** |
| LmjF24.1660 | hypothetical protein, conserved | -1,73 |
| **LmjF24.1900** | **hypothetical protein, conserved** | **-2,07** |
| **LmjF24.2040** | **hypothetical protein, conserved** | **-3,08** |
| **LmjF25.0240** | **hypothetical protein, conserved** | **-1,70** |
| **LmjF25.0350** | **hypothetical protein, conserved** | **-2,06** |
| LmjF25.0610 | hypothetical protein, conserved | -1,90 |
| **LmjF25.0950** | **hypothetical protein, conserved** | **-2,54** |
| LmjF25.1000 | hypothetical protein, conserved | -1,81 |
| **LmjF25.1670** | **rev7, putative** | **-2,68** |
| **LmjF25.1690** | **hypothetical protein, conserved** | **-2,01** |
| **LmjF25.1740** | **hypothetical protein, conserved** | **-1,99** |
| LmjF25.1750 | hypothetical protein, conserved | -2,20 |
| **LmjF25.1910** | **hypothetical protein, conserved** | **-2,31** |
| LmjF26.0390 | RNA editing 3' terminal uridylyl transferase 2 | -2,32 |
| LmjF26.0680 | hypothetical protein, conserved | -1,79 |
| **LmjF26.0780** | **hypothetical protein, conserved** | **-2,13** |
| **LmjF26.0940** | **hypothetical protein, conserved** | **-2,21** |
| **LmjF26.1110** | **hypothetical protein, conserved** | **-2,22** |
| **LmjF26.1450** | **hypothetical protein, conserved** | **-2,73** |
| **LmjF26.1490** | **hypothetical protein, unknown function** | **-2,77** |
| **LmjF26.1500** | **hypothetical protein, unknown function** | **-1,87** |
| LmjF26.1720 | hypothetical protein, conserved | -1,69 |
| **LmjF26.1830** | **hypothetical protein, conserved** | **-1,72** |
| LmjF26.1830 | hypothetical protein, conserved | -1,87 |
| **LmjF26.1980** | **hypothetical protein, conserved** | **-2,81** |
| **LmjF26.2260** | **syntaxin binding protein 1, putative** | **-2,25** |
| LmjF26.2300 | hypothetical protein, conserved | -2,04 |
| **LmjF26.2550** | **hypothetical protein, conserved** | **-2,00** |
| **LmjF26.2570** | **protein kinase, putative** | **-3,68** |
| **LmjF26.2670** | **p-glycoprotein-like protein** | **-3,58** |
| **LmjF26.2680** | **hypothetical protein, unknown function** | **-22,13** |
| LmjF27.0400 | hypothetical protein, conserved | -2,48 |
| LmjF27.0510 | calpain-like cysteine peptidase, putative | -2,15 |
| LmjF27.0540 | hypothetical protein, conserved | -1,91 |
| LmjF27.0850 | hypothetical protein, conserved | -1,76 |
| LmjF27.1480 | hypothetical protein, conserved | -2,35 |
| **LmjF27.1950** | **hypothetical protein, conserved** | **-2,64** |
| LmjF27.2020 | d-lactate dehydrogenase-like protein | -2,05 |
| **LmjF27.2460** | **protein kinase, putative** | **-1,99** |
| LmjF27.2620 | hypothetical protein, conserved | -1,72 |
| LmjF28.0060 | hypothetical protein, conserved | -1,88 |
| **LmjF28.0220** | **hypothetical protein, conserved** | **-1,93** |
| **LmjF28.0370** | **hypothetical protein, unknown function** | **-2,96** |
| LmjF28.0400 | katanin, putative | -1,81 |
| LmjF28.0570 | major surface protease gp63, putative | -2,36 |
| LmjF28.1515 | hypothetical protein, conserved | -1,71 |
| LmjF28.1530 | DEAD box RNA helicase, putative | -1,72 |
| **LmjF28.1710** | **hypothetical protein, conserved** | **-1,89** |
| **LmjF28.1770** | **hypothetical protein, conserved** | **-1,70** |
| **LmjF28.2210** | **glycoprotein 96-92, putative** | **-2,25** |
| **LmjF28.2300** | **hypothetical protein, conserved** | **-2,27** |
| **LmjF28.2490** | **hypothetical protein, conserved** | **-2,25** |
| LmjF28.2565 | hypothetical protein, conserved | -1,70 |
| **LmjF28.2920** | **hypothetical protein, conserved** | **-1,81** |
| **LmjF28.2960** | **hypothetical protein, conserved** | **-1,85** |
| **LmjF29.0090** | **GTP-binding protein-like protein** | **-1,81** |
| LmjF29.0350 | hypothetical protein, conserved | -1,69 |
| **LmjF29.0570** | **hypothetical protein, conserved** | **-2,19** |
| LmjF29.0730 | hypothetical protein, conserved | -1,83 |
| **LmjF29.1000** | **hypothetical protein, conserved** | **-2,31** |
| LmjF29.1030 | hypothetical protein, conserved | -2,20 |
| LmjF29.1360 | RNA binding protein, putative | -1,77 |
| LmjF29.1420 | hypothetical protein, conserved | -2,63 |
| **LmjF29.1440** | **clathrin coat assembly protein AP19, putative** | **-1,82** |
| LmjF29.1450 | phosphatidylinositol-kinase domain protein, putative | -1,75 |
| **LmjF29.1470** | **hypothetical protein, conserved** | **-2,68** |
| **LmjF29.1570** | **glutamamyl carboxypeptidase, putative** | **-3,93** |
| **LmjF29.1710** | **MutS-like protein** | **-2,53** |
| **LmjF29.1740** | **histone H2A, putative** | **-2,81** |
| LmjF29.1770 | paraflagellar rod protein 1D, putative | -1,80 |
| **LmjF29.1920** | **hypothetical protein, unknown function** | **-2,05** |
| **LmjF29.2030** | **N-acetylglucosaminyl transferase component, putative** | **-3,04** |
| **LmjF29.2410** | **hypothetical protein, conserved** | **-1,86** |
| **LmjF29.2480** | **hypothetical protein, conserved** | **-2,40** |
| **LmjF29.2590** | **hypothetical protein, conserved** | **-1,94** |
| **LmjF29.2720** | **protein kinase, putative** | **-1,75** |
| LmjF29.2840 | hypothetical protein, conserved | -1,69 |
| LmjF30.0390 | hypothetical protein, unknown function | -1,95 |
| **LmjF30.0840** | **hypothetical protein, conserved** | **-1,84** |
| **LmjF30.1050** | **mitochondrial carrier protein-like protein** | **-3,26** |
| **LmjF30.1170** | **hypothetical protein, unknown function** | **-2,29** |
| LmjF30.1330 | ABC transporter, putative | -2,37 |
| LmjF30.1400 | hypothetical protein, conserved | -1,76 |
| **LmjF30.1985** | **hypothetical protein, conserved** | **-2,97** |
| **LmjF30.2420** | **hypothetical protein, conserved** | **-1,75** |
| LmjF30.2620 | hypothetical protein, conserved | -1,74 |
| **LmjF30.2650** | **hypothetical protein, conserved** | **-2,10** |
| **LmjF30.2790** | **hypothetical protein, conserved** | **-1,79** |
| LmjF30.2850 | hypothetical protein, conserved | -1,87 |
| **LmjF30.2920** | **hypothetical protein, conserved** | **-3,78** |
| LmjF30.3700 | hypothetical protein, conserved | -1,80 |
| LmjF31.0350 | amino acid transporter aATP11, putative | -1,84 |
| **LmjF31.0410** | **calpain-like cysteine peptidase, putative** | **-1,73** |
| **LmjF31.0810** | **hypothetical protein, conserved** | **-2,77** |
| **LmjF31.1340** | **hypothetical protein, conserved** | **-2,50** |
| **LmjF31.1440** | **hypothetical protein, unknown function** | **-5,74** |
| **LmjF31.1760** | **hypothetical protein, unknown function** | **-1,88** |
| **LmjF31.1810** | **acetylornithine deacetylase-like protein** | **-4,09** |
| LmjF31.2200 | hypothetical protein, unknown function | -1,68 |
| LmjF31.2240 | hypothetical protein, unknown function | -1,78 |
| **LmjF31.2320** | **helicase-like protein** | **-1,71** |
| LmjF31.2380 | hypothetical protein, unknown function | -1,87 |
| **LmjF31.2400** | **hypothetical protein, unknown function** | **-2,56** |
| **LmjF32.0810** | **serine/threonine protein kinase, putative** | **-3,20** |
| **LmjF32.0930** | **hypothetical protein, conserved** | **-3,25** |
| LmjF32.0970 | calpain-like cysteine peptidase, putative | -1,96 |
| LmjF32.1240 | hypothetical protein, conserved | -1,68 |
| LmjF32.1260 | hypothetical protein, conserved | -4,74 |
| LmjF32.1300 | polypeptide deformylase-like protein, putative | -2,51 |
| LmjF32.1450 | hypothetical protein, conserved | -1,83 |
| LmjF32.1680 | hypothetical protein, conserved | -2,33 |
| LmjF32.1760 | hypothetical protein, conserved | -2,25 |
| LmjF32.1780 | hypothetical protein, conserved | -2,31 |
| LmjF32.1910 | hypothetical protein, conserved | -1,86 |
| LmjF32.1920 | hypothetical protein, conserved | -1,73 |
| LmjF32.1965 | hypothetical protein | -2,60 |
| **LmjF32.2210** | **hypothetical protein, conserved** | **-2,20** |
| LmjF32.2270 | membrane associated protein-like protein | -1,76 |
| LmjF32.2280 | hypothetical protein, conserved | -1,70 |
| LmjF32.2400 | hypothetical protein, conserved | -1,73 |
| LmjF32.2740 | hypothetical protein, conserved | -1,77 |
| LmjF32.2840 | hypothetical protein, conserved | -1,69 |
| **LmjF32.3070** | **hypothetical protein, conserved** | **-2,39** |
| LmjF32.3100 | hypothetical protein, unknown function | -1,81 |
| **LmjF32.3150** | **hypothetical protein, conserved** | **-1,83** |
| LmjF32.3450 | hypothetical protein, conserved | -1,84 |
| LmjF32.3555 | hypothetical protein, conserved | -2,11 |
| LmjF32.3670 | 3-hydroxyisobutyryl-coenzyme a hydrolase-like protein | -3,00 |
| LmjF32.3730 | hypothetical protein, conserved | -2,02 |
| LmjF32.3760 | hypothetical protein, conserved | -2,21 |
| **LmjF32.3790** | **cyclin-dependent kinase regulatory subunit** | **-1,80** |
| LmjF32.3870 | myosin heavy chain, putative | -1,78 |
| LmjF32.3930 | hypothetical protein, conserved | -1,78 |
| **LmjF32.3960** | **hypothetical protein, conserved** | **-2,58** |
| LmjF33.0080 | hypothetical protein, conserved | -1,74 |
| **LmjF33.0160** | **hypothetical protein, unknown function** | **-2,03** |
| LmjF33.0220 | hypothetical protein, conserved | -2,44 |
| **LmjF33.0290** | **glucose transporter/membrane transporter D2, putative** | **-2,46** |
| **LmjF33.0500** | **hypothetical protein, conserved** | **-2,42** |
| LmjF33.0520 | d-xylulose reductase, putative | -1,72 |
| **LmjF33.0610** | **hypothetical protein, conserved** | **-2,13** |
| **LmjF33.0820** | **beta tubulin** | **-2,28** |
| **LmjF33.0880** | **hypothetical protein, conserved** | **-2,30** |
| **LmjF33.0930** | **hypothetical protein, conserved** | **-2,79** |
| **LmjF33.1080** | **hypothetical protein, conserved** | **-1,86** |
| LmjF33.1250 | hypothetical protein, conserved | -2,51 |
| **LmjF33.1410** | **hypothetical protein, conserved** | **-2,47** |
| LmjF33.1530 | hypothetical protein, conserved | -2,43 |
| **LmjF33.1830** | **protein kinase, putative** | **-2,28** |
| **LmjF33.2890** | **hypothetical protein, conserved** | **-2,30** |
| **LmjF33.2900** | **hypothetical protein, unknown function** | **-2,28** |
| **LmjF33.2910** | **hypothetical protein, conserved** | **-3,15** |
| **LmjF33.2930** | **hypothetical protein, conserved** | **-3,53** |
| LmjF33.2940 | hypothetical protein, conserved | -2,18 |
| LmjF33.2960 | hypothetical protein, conserved | -2,73 |
| LmjF33.2980 | hypothetical protein, conserved | -2,19 |
| **LmjF33.3000** | **hypothetical protein, unknown function** | **-1,76** |
| LmjF33.3010 | hypothetical protein, conserved | -1,80 |
| LmjF33.3020 | hypothetical protein, unknown function | -1,83 |
| LmjF34.0190 | hypothetical protein, conserved | -2,43 |
| LmjF34.0280 | calpain-like cysteine peptidase, putative | -2,13 |
| **LmjF34.0410** | **hypothetical protein, conserved** | **-2,15** |
| **LmjF34.0460** | **hypothetical protein, unknown function** | **-2,99** |
| **LmjF34.0850** | **serine/threonine-protein phosphatase PP1, putative** | **-2,35** |
| **LmjF34.1350** | **hypothetical protein, conserved** | **-2,60** |
| **LmjF34.1380** | **hypothetical protein, unknown function** | **-2,51** |
| **LmjF34.1490** | **hypothetical protein, conserved** | **-3,01** |
| LmjF34.1520 | hypothetical protein, conserved | -1,91 |
| LmjF34.1520 | hypothetical protein, conserved | -2,18 |
| **LmjF34.1960** | **amastin-like surface protein, putative** | **-1,98** |
| **LmjF34.2030** | **hypothetical protein, conserved** | **-1,86** |
| LmjF34.2080 | hypothetical protein, conserved | -1,91 |
| **LmjF34.2190** | **dual specificity protein phosphatase, putative** | **-2,69** |
| LmjF34.2310 | hypothetical protein, conserved | -1,70 |
| **LmjF34.2370** | **hypothetical protein, conserved** | **-2,73** |
| **LmjF34.2765** | **hypothetical protein, conserved** | **-3,64** |
| LmjF34.3120 | lipophosphoglycan biosynthetic protein (lpg2) | -1,70 |
| **LmjF34.3320** | **hypothetical protein, conserved** | **-2,01** |
| LmjF34.3330 | cytochrome p450-like protein | -2,05 |
| LmjF34.3350 | ATPase-like protein | -1,88 |
| LmjF34.3370 | hypothetical protein, conserved | -1,83 |
| LmjF34.3390 | hypothetical protein, conserved | -1,86 |
| LmjF34.3400 | hypothetical protein, unknown function | -1,75 |
| **LmjF34.3710** | **hypothetical protein, conserved** | **-2,10** |
| LmjF34.3745 | hypothetical protein | -1,69 |
| **LmjF34.3770** | **hypothetical protein, conserved** | **-2,72** |
| LmjF34.3880 | dynein heavy chain, putative | -1,72 |
| LmjF34.4040 | hypothetical protein, conserved | -1,73 |
| **LmjF34.4120** | **hypothetical protein, conserved** | **-2,27** |
| LmjF34.4240 | hypothetical protein, conserved | -1,98 |
| **LmjF34.4540** | **hypothetical protein, conserved** | **-1,85** |
| **LmjF34.4620** | **hypothetical protein, conserved** | **-2,11** |
| **LmjF35.0430** | **hypothetical protein, conserved** | **-1,78** |
| LmjF35.0440 | hypothetical protein, unknown function | -2,17 |
| **LmjF35.0480** | **hypothetical protein, conserved** | **-1,73** |
| **LmjF35.0500** | **proteophosphoglycan ppg3, putative** | **-3,72** |
| **LmjF35.0540** | **proteophosphoglycan 5** | **-4,19** |
| **LmjF35.0550** | **proteophosphoglycan ppg1** | **-2,21** |
| LmjF35.0610 | hypothetical protein, conserved | -1,73 |
| **LmjF35.0740** | **hypothetical protein, conserved** | **-1,93** |
| LmjF35.1100 | hypothetical protein, conserved | -1,70 |
| **LmjF35.1360** | **hypothetical protein, unknown function** | **-2,29** |
| LmjF35.1490 | hypothetical protein, conserved | -1,82 |
| LmjF35.1510 | hypothetical protein, conserved | -1,92 |
| **LmjF35.1620** | **hypothetical protein, conserved** | **-2,20** |
| LmjF35.1940 | hypothetical protein, unknown function | -1,80 |
| LmjF35.1980 | hypothetical protein, conserved | -1,82 |
| LmjF35.2580 | hypothetical protein, conserved | -2,00 |
| LmjF35.2740 | galactokinase-like protein | -2,58 |
| **LmjF35.2790** | **hypothetical protein, conserved** | **-2,63** |
| LmjF35.2850 | hypothetical protein, conserved | -1,68 |
| LmjF35.3040 | hypothetical protein, conserved | -1,83 |
| LmjF35.3120 | hypothetical protein, conserved | -1,72 |
| **LmjF35.3300** | **hypothetical protein, unknown function** | **-1,97** |
| LmjF35.3320 | transport protein particle (TRAPP) subunit, putative | -1,69 |
| **LmjF35.3530** | **hypothetical protein, unknown function** | **-2,41** |
| **LmjF35.3550** | **hypothetical protein, conserved** | **-1,99** |
| **LmjF35.3660** | **hypothetical protein, conserved** | **-2,78** |
| LmjF35.3690 | hypothetical protein, conserved | -1,81 |
| **LmjF35.3900** | **hypothetical protein, conserved** | **-1,76** |
| **LmjF35.4045** | **hypothetical protein, conserved** | **-2,07** |
| **LmjF35.4170** | **hypothetical protein, conserved** | **-2,01** |
| **LmjF35.4230** | **hypothetical protein, conserved** | **-2,38** |
| LmjF35.4480 | hypothetical protein, conserved | -2,09 |
| **LmjF35.4610** | **hypothetical protein, conserved** | **-2,33** |
| LmjF35.4690 | hypothetical protein, conserved | -1,74 |
| LmjF35.4800 | AMP deaminase, putative | -1,74 |
| LmjF35.5190 | NIMA-related kinase, putative | -1,74 |
| **LmjF35.5220** | **hypothetical protein, conserved** | **-2,18** |
| LmjF35.5340 | hypothetical protein, conserved | -1,98 |
| LmjF36.0090 | hypothetical protein, conserved | -2,22 |
| LmjF36.0110 | hypothetical protein, conserved | -1,70 |
| LmjF36.0160 | hypothetical protein, conserved | -1,88 |
| **LmjF36.0230** | **peptidyl-prolyl cis-trans isomerase, putative** | **-1,76** |
| **LmjF36.0280** | **hypothetical protein, conserved** | **-2,55** |
| **LmjF36.0420** | **amino acid permease-like protein** | **-2,94** |
| LmjF36.0450 | hypothetical protein, conserved | -1,81 |
| LmjF36.1360 | adenylate kinase, putative | -2,09 |
| LmjF36.1400 | hypothetical protein, conserved | -1,70 |
| **LmjF36.1450** | **hypothetical protein, conserved** | **-2,90** |
| LmjF36.1595 | hypothetical protein, conserved | -1,95 |
| **LmjF36.1680** | **hypothetical protein, conserved** | **-1,80** |
| **LmjF36.2090** | **hypothetical protein, conserved** | **-2,75** |
| LmjF36.2290 | protein kinase, putative | -1,81 |
| **LmjF36.2550** | **hypothetical protein, conserved** | **-2,00** |
| LmjF36.2760 | hypothetical protein, conserved | -3,01 |
| **LmjF36.2890** | **ATP-binding cassette protein, putative** | **-2,83** |
| LmjF36.3360 | hypothetical protein, conserved | -1,80 |
| **LmjF36.3620** | **hypothetical protein, conserved** | **-2,13** |
| **LmjF36.3920** | **hypothetical protein, conserved** | **-1,73** |
| **LmjF36.4130** | **hypothetical protein, unknown function** | **-2,35** |
| **LmjF36.4140** | **hypothetical protein, unknown function** | **-3,84** |
| **LmjF36.4145** | **transcription factor S-II-like protein** | **-2,50** |
| LmjF36.4230 | hypothetical protein, conserved | -2,80 |
| **LmjF36.4530** | **ABC1 protein, putative** | **-2,30** |
| LmjF36.4780 | hypothetical protein, conserved | -1,84 |
| **LmjF36.5210** | **hypothetical protein, conserved** | **-2,20** |
| **LmjF36.5340** | **hypothetical protein, conserved** | **-1,98** |
| LmjF36.5410 | selenophosphate synthetase, putative | -1,69 |
| LmjF36.5480 | hypothetical protein, conserved | -2,00 |
| **LmjF36.5630** | **hypothetical protein, conserved** | **-2,10** |
| LmjF36.5790 | hypothetical protein, conserved | -1,81 |
| LmjF36.5830 | hypothetical protein, conserved | -1,93 |
| **LmjF36.5870** | **hypothetical protein, conserved** | **-2,45** |
| LmjF36.6030 | hypothetical protein, conserved | -1,97 |
| **LmjF36.6110** | **centrin, putative** | -1,73 |
| LmjF36.6180 | hypothetical protein, conserved | -1,75 |
| LmjF36.6200 | hypothetical protein, conserved | -1,89 |
| **LmjF36.6290** | **glucose transporter, lmgt2, putative** | **-3,10** |
| **LmjF36.6300** | **glucose transporter, putative** | **-2,56** |
| **LmjF36.6430** | **protein transport protein sec23-like protein** | **-2,26** |
| **LmjF36.6480** | **histidine secretory acid phosphatase, putative** | **-2,66** |
| LmjF36.6480 | histidine secretory acid phosphatase, putative | -2,93 |
| LmjF36.6480 | histidine secretory acid phosphatase, putative | -3,04 |
| **LmjF36.6530** | **hypothetical protein, conserved** | **-1,91** |
| **Upregulated genes** | | |
| LmjF01.0030 | MCAK-like kinesin, putative | 1,75 |
| LmjF01.0150 | hypothetical protein, conserved | 2,23 |
| LmjF01.0170 | hypothetical protein, conserved | 1,79 |
| LmjF01.0180 | CLC-type chloride channel, putative | 1,71 |
| LmjF01.0290 | hypothetical protein, conserved | 1,91 |
| LmjF01.0320 | poly(A) export protein, putative | 2,16 |
| LmjF01.0340 | hypothetical protein, conserved | 2,05 |
| LmjF01.0350 | hypothetical protein, conserved | 1,80 |
| LmjF01.0660 | hypothetical protein, conserved | 2,61 |
| **LmjF01.0780** | **eukaryotic initiation factor 4a, putative** | **1,96** |
| LmjF01.0820 | potassium channel subunit-like protein | 1,72 |
| **LmjF02.0200** | **phosphoglycan beta 1,3 galactosyltransferase-like protein** | **2,16** |
| **LmjF02.0460** | **hypothetical protein, conserved** | **1,98** |
| **LmjF02.0660** | **hypothetical protein, conserved** | **1,83** |
| **LmjF03.0030** | **D-3-phosphoglycerate dehydrogenase-like protein** | **2,59** |
| LmjF03.0090 | DNA primase large subunit, putative | 1,83 |
| **LmjF03.0200** | **delta-1-pyrroline-5-carboxylate dehydrogenase, putative** | **2,07** |
| **LmjF03.0310** | **hypothetical protein** | **2,51** |
| **LmjF03.0440** | **60S acidic ribosomal protein P2, putative** | **1,87** |
| **LmjF03.0460** | **hypothetical protein** | **2,07** |
| LmjF03.0480 | hypothetical protein, conserved | 1,70 |
| LmjF03.0670 | hypothetical protein, conserved | 1,80 |
| LmjF03.0820 | hypothetical protein, conserved | 1,73 |
| **LmjF03.0840** | **hypothetical protein, conserved** | **1,91** |
| **LmjF04.0040** | **hypothetical protein** | **2,08** |
| LmjF04.0310 | beta-fructofuranosidase, putative | 1,90 |
| LmjF04.0580 | spermidine synthase, putative | 2,15 |
| LmjF04.0710 | hypothetical protein, conserved | 1,76 |
| **LmjF05.0080** | **hypothetical protein, conserved** | **1,69** |
| **LmjF05.0350** | **trypanothione reductase** | **2,01** |
| **LmjF05.0400** | **structural maintenance of chromosome (SMC), putative** | **2,13** |
| **LmjF05.0460** | **GTPase, putative** | **2,17** |
| **LmjF05.1000** | **hypothetical protein, conserved** | **1,97** |
| **LmjF05.1110** | **hypothetical protein, conserved** | **3,80** |
| LmjF06.0050 | hypothetical protein, conserved | 1,79 |
| LmjF06.0140 | proteasome beta 6 subunit, putative | 2,09 |
| LmjF06.0200 | hypothetical protein, conserved | 1,77 |
| **LmjF06.0560** | **deoxyuridine triphosphatase, putative** | **1,93** |
| **LmjF06.0610** | **carbonic anhydrase family protein, putative** | **2,49** |
| LmjF06.0700 | hypothetical protein, conserved | 1,95 |
| **LmjF06.0860** | **dihydrofolate reductase-thymidylate synthase** | **3,37** |
| **LmjF06.0930** | **2,4-dienoyl-coa reductase-like protein** | **2,76** |
| LmjF06.1010 | hypothetical protein, conserved | 2,24 |
| LmjF07.0025 | hypothetical protein, conserved | 2,49 |
| LmjF07.0040 | hypothetical protein, conserved | 1,85 |
| LmjF07.0350 | hypothetical protein, conserved | 1,80 |
| **LmjF07.0470** | **hypothetical protein, conserved** | **2,19** |
| LmjF07.0510 | 60S ribosomal protein L7a, putative | 1,99 |
| **LmjF07.0710** | **centrin, putative** | **3,00** |
| **LmjF07.0802** | **hypothetical protein, conserved** | **2,10** |
| LmjF07.0820 | hypothetical protein, unknown function | 2,31 |
| **LmjF07.0860** | **hypothetical protein, unknown function** | **3,06** |
| LmjF07.1140 | peptide methionine sulfoxide reductase-like | 1,82 |
| LmjF08.0030 | vesicle-associated membrane protein, putative | 2,40 |
| LmjF08.0050 | hypothetical protein, unknown function | 1,80 |
| **LmjF08.0170** | **hypothetical protein, unknown function** | **2,58** |
| LmjF08.0760 | amastin-like protein | 4,27 |
| LmjF08.0770 | amastin-like protein | 3,18 |
| LmjF08.0950 | hypothetical protein, conserved | 1,75 |
| LmjF08.1140 | hypothetical protein, conserved | 1,72 |
| **LmjF09.0400** | **protein kinase, putative** | **2,73** |
| LmjF09.1030 | hypothetical protein, conserved | 1,70 |
| LmjF09.1370 | hypothetical protein, conserved | 2,26 |
| LmjF10.0100 | hypothetical protein, conserved | 2,08 |
| LmjF10.0150 | hypothetical protein, conserved | 1,90 |
| LmjF10.0210 | nucleolar protein, putative | 1,86 |
| **LmjF10.0220** | **hypothetical protein, unknown function** | **1,82** |
| LmjF10.0300 | hypothetical protein, conserved | 2,27 |
| LmjF10.0320 | hypothetical protein, conserved | 1,98 |
| LmjF10.0400 | pteridine transporter ft5, putative | 1,77 |
| LmjF10.0480 | GP63, leishmanolysin | 3,09 |
| LmjF10.0580 | hypothetical protein, conserved | 1,69 |
| LmjF10.0600 | hypothetical protein, conserved | 1,75 |
| LmjF10.0670 | hypothetical protein, conserved | 1,91 |
| LmjF10.0720 | transmembrane amino acid transporter protein-like protein | 2,17 |
| LmjF10.0740 | hypothetical protein, conserved | 1,92 |
| LmjF10.0750 | hypothetical protein, conserved | 1,87 |
| LmjF10.0810 | hypothetical protein, conserved | 1,69 |
| **LmjF10.0860** | **hypothetical protein, conserved** | **2,34** |
| LmjF10.0980 | hypothetical protein, unknown function | 1,80 |
| LmjF10.1030 | RNA-binding protein-like protein | 1,94 |
| LmjF10.1200 | hypothetical protein, conserved | 1,81 |
| LmjF10.1230 | hypothetical protein, conserved | 2,11 |
| LmjF10.1270 | hypothetical protein, conserved | 2,56 |
| **LmjF10.1290** | **hypothetical protein, conserved** | **3,21** |
| LmjF10.1300 | phosphate-repressible phosphate permease-like protein | 2,86 |
| LmjF10.1320 | fatty acid desaturase, putative | 2,64 |
| LmjF11.0330 | PIF1 helicase-like protein, putative | 1,90 |
| **LmjF11.0470** | **pumilio-repeat, RNA-binding protein, putative** | **1,89** |
| **LmjF11.0650** | **hypothetical protein, conserved** | **2,42** |
| LmjF11.0780 | 40S ribosomal protein S21, putative | 1,81 |
| LmjF11.0820 | hypothetical protein, conserved | 1,69 |
| LmjF11.0860 | hypothetical protein, conserved | 1,82 |
| LmjF11.0970 | 40S ribosomal protein S5 | 2,31 |
| **LmjF12.0310** | **hypothetical protein, conserved** | **3,33** |
| **LmjF12.0350** | **hypothetical protein, conserved** | **2,96** |
| **LmjF12.0400** | **3'-nucleotidase/nuclease, putative** | **2,05** |
| **LmjF12.0480** | **hypothetical protein, unknown function** | **2,01** |
| **LmjF12.0490** | **hypothetical protein, unknown function** | **2,21** |
| **LmjF12.0670** | **cytochrome c oxidase subunit iv** | **1,72** |
| LmjF13.0200 | hypothetical protein, unknown function | 2,48 |
| LmjF13.0220 | small Rab GTP binding protein, putative | 1,92 |
| **LmjF13.0450** | **hypothetical protein, conserved** | **3,50** |
| LmjF13.0470 | hypothetical protein, conserved | 1,73 |
| LmjF13.0570 | 40S ribosomal protein S12, putative | 2,05 |
| LmjF13.0700 | kinesin, putative | 2,02 |
| LmjF13.0870 | mitochondrial processing peptidase alpha subunit, putative | 1,89 |
| LmjF13.1020 | hypothetical protein, unknown function | 1,98 |
| LmjF13.1030 | hypothetical protein, conserved | 3,00 |
| **LmjF13.1210** | **NT3, nucleobase/nucleoside transporter 8.1, putative** | **13,32** |
| LmjF13.1230 | 40S ribosomal protein S4, putative | 2,32 |
| LmjF13.1240 | hypothetical protein, conserved | 1,75 |
| LmjF13.1280 | XPA-interacting protein, putative | 1,68 |
| LmjF13.1360 | hypothetical protein, conserved | 1,85 |
| LmjF13.1370 | hypothetical protein, conserved | 1,73 |
| **LmjF13.1620** | **squalene monooxygenase-like protein** | **1,83** |
| **LmjF13.1630** | **mitochondrial DNA polymerase I protein D, putative** | **2,38** |
| LmjF13.1660 | chaperonin TCP20, putative | 2,28 |
| LmjF13.1680 | pyrroline-5-carboxylate reductase | 2,52 |
| **LmjF13.1690** | **hypothetical protein, conserved** | **6,58** |
| LmjF14.0180 | carboxypeptidase, putative | 2,26 |
| LmjF14.0980 | hypothetical protein, conserved | 2,05 |
| LmjF14.1490 | synaptojanin (N-terminal domain), putative | 1,71 |
| **LmjF15.0230** | **lysyl-tRNA synthetase, putative** | **2,00** |
| **LmjF15.0270** | **replication Factor A 28 kDa subunit, putative** | **2,22** |
| **LmjF15.0520** | **ecotin, putative** | **2,38** |
| LmjF15.0850 | hypothetical protein, conserved | 1,72 |
| LmjF15.1207 | 60S acidic ribosomal protein P2 | 2,15 |
| **LmjF15.1350** | **hypothetical protein, conserved** | **1,73** |
| **LmjF15.1470** | **ribosomal protein S6, putative** | **1,83** |
| LmjF15.1490 | hypothetical protein, conserved | 2,03 |
| **LmjF15.1520** | **hypothetical protein, conserved** | **2,22** |
| LmjF15.1560 | condensin subunit 1, putative | 1,95 |
| LmjF16.0050 | hypothetical protein, conserved | 1,80 |
| LmjF16.0140 | eukaryotic translation initiation factor 1A, putative | 1,72 |
| **LmjF16.0420** | **hypothetical protein, conserved** | **1,99** |
| LmjF16.0490 | hypothetical protein, conserved | 2,30 |
| LmjF16.0500 | hypothetical protein, unknown function | 3,31 |
| **LmjF16.0520** | **hypothetical protein, conserved** | **2,27** |
| LmjF16.0590 | carbamoyl-phosphate synthase, putative | 1,83 |
| LmjF16.0700 | hypothetical protein, conserved | 1,75 |
| LmjF16.0760 | transaldolase, putative | 1,74 |
| LmjF16.0860 | hypothetical protein, conserved | 1,80 |
| LmjF16.0890 | hypothetical protein, conserved | 2,39 |
| LmjF16.0960 | hypothetical protein, conserved | 1,88 |
| LmjF16.1090 | hypothetical protein, unknown function | 1,81 |
| LmjF16.1170 | 60S ribosomal protein L39, putative | 2,28 |
| LmjF16.1320 | cytochrome c, putative | 1,93 |
| LmjF16.1330 | hypothetical protein, conserved | 1,71 |
| LmjF16.1420 | hypothetical protein, conserved | 2,09 |
| LmjF16.1540 | DNA polymerase I alpha catalytic subunit, putative | 2,75 |
| LmjF16.1580 | kinesin, putative | 1,78 |
| LmjF16.1600 | hypothetical protein, conserved | 2,16 |
| **LmjF17.0340** | **hypothetical protein, conserved** | **1,71** |
| **LmjF17.0390** | **protein kinase, putative** | **1,83** |
| **LmjF17.0430** | **hypothetical protein, conserved** | **2,29** |
| **LmjF17.0690** | **hypothetical protein, conserved** | **2,93** |
| **LmjF17.0790** | **protein kinase, putative** | **2,14** |
| LmjF17.1010 | hydrolase, alpha/beta fold family-like protein | 2,79 |
| LmjF17.1440 | hypothetical protein, unknown function | 1,96 |
| **LmjF18.0020** | **diphosphomevalonate decarboxylase, putative** | **1,97** |
| **LmjF18.0190** | **hypothetical protein, unknown function** | **1,95** |
| LmjF18.0230 | 60S ribosomal protein L7, putative | 1,70 |
| **LmjF18.0280** | **hypothetical protein, conserved** | **1,98** |
| **LmjF18.0300** | **hypothetical protein, conserved** | **2,35** |
| **LmjF18.0430** | **hypothetical protein, unknown function** | **2,75** |
| LmjF18.0440 | phosphatidic acid phosphatase, putative | 2,33 |
| LmjF18.0510 | aconitase, putative | 1,76 |
| LmjF18.0940 | hypothetical protein, conserved | 1,92 |
| **LmjF18.1060** | **calpain-like cysteine peptidase, putative** | **2,21** |
| LmjF18.1120 | hypothetical protein, conserved | 1,92 |
| LmjF18.1370 | heat shock protein, putative | 1,92 |
| LmjF18.1410 | hypothetical protein, conserved | 1,71 |
| LmjF18.1580 | nonspecific nucleoside hydrolase | 2,12 |
| **LmjF19.0060** | **40S ribosomal protein S2** | **2,11** |
| **LmjF19.0100** | **fibrillarin, putative** | **1,74** |
| **LmjF19.0240** | **hypothetical protein, conserved** | **3,11** |
| **LmjF19.0800** | **ABC transporter, putative** | **3,97** |
| LmjF19.1020 | tRNA pseudouridine synthase A-like protein | 1,80 |
| LmjF19.1210 | hypothetical protein, conserved | 1,75 |
| LmjF19.1420 | cysteine protease A | 1,70 |
| **LmjF20.0030** | **hypothetical protein, conserved** | **3,29** |
| **LmjF20.0050** | **anti-silencing protein asf 1-like protein** | **1,87** |
| **LmjF20.0140** | **hypothetical protein, conserved** | **2,11** |
| LmjF20.0220 | hypothetical protein, conserved | 1,88 |
| **LmjF20.0460** | **hypothetical protein, conserved** | **2,99** |
| LmjF20.0520 | hypothetical protein, conserved | 1,86 |
| **LmjF20.0700** | **hypothetical protein, conserved** | **1,77** |
| LmjF20.0830 | phosphopantetheinyl transferase-like protein | 2,32 |
| LmjF20.0850 | hypothetical protein, conserved | 1,92 |
| **LmjF20.1070** | **hypothetical protein, unknown function** | **2,04** |
| LmjF20.1660 | hypothetical protein, conserved | 1,95 |
| **LmjF20.1670** | **ribosome biogenesis protein, putative** | **1,71** |
| **LmjF20.1700** | **hypothetical protein, conserved** | **1,77** |
| LmjF21.0090 | hypothetical protein, conserved | 1,80 |
| LmjF21.0125 | DNA topoisomerase 1A | 1,88 |
| **LmjF21.0440** | **ubiquitin-conjugating enzyme-like protein** | **3,53** |
| **LmjF21.0540** | **la RNA binding protein, putative** | **1,76** |
| **LmjF21.0710** | **ribonuclease L inhibitor, putative** | **2,39** |
| LmjF21.0750 | hypothetical protein, conserved | 1,99 |
| LmjF21.1040 | kinesin, putative | 1,77 |
| LmjF21.1070 | 40S ribosomal protein S23, putative | 2,18 |
| **LmjF21.1090** | **t-complex protein 1, delta subunit, putative** | **1,95** |
| LmjF21.1210 | thymidine kinase, putative | 1,86 |
| LmjF21.1500 | hypothetical protein, conserved | 1,96 |
| **LmjF21.1560** | **hypothetical protein, conserved** | **2,43** |
| LmjF21.1660 | mitochondrial structure specific endonuclease I (SSE-1), putative | 1,76 |
| LmjF21.1710 | cytochrome C oxidase subunit VI, putative | 1,80 |
| **LmjF21.1730** | **hypothetical protein, conserved** | **1,69** |
| LmjF21.1760 | centromere/microtubule binding protein cbf5, putative | 2,27 |
| **LmjF22.0170** | **hypothetical protein, conserved** | **2,46** |
| **LmjF22.0225** | **hypothetical protein, conserved** | **4,62** |
| LmjF22.0230 | amino acid permease, putative | 2,07 |
| LmjF22.0240 | hypothetical protein, unknown function | 1,76 |
| LmjF22.0490 | protein kinase, putative | 1,69 |
| **LmjF22.0600** | **hypothetical protein, conserved** | **2,26** |
| LmjF22.0740 | hypothetical protein, conserved | 2,64 |
| **LmjF22.0790** | **hypothetical protein, conserved** | **1,86** |
| LmjF22.1560 | 40S ribosomal protein L14, putative | 1,71 |
| LmjF22.1600 | Ser/Thr protein phosphatase, putative | 2,15 |
| **LmjF23.0010** | **hypothetical protein, conserved** | **2,44** |
| LmjF23.0020 | hypothetical protein, conserved | 2,72 |
| LmjF23.0025 | hypothetical protein, conserved | 2,53 |
| **LmjF23.0030** | **beta propeller protein, putative** | **5,56** |
| **LmjF23.0040** | **peroxidoxin** | **3,33** |
| **LmjF23.0050** | **cyclophilin, putative** | **5,32** |
| LmjF23.0060 | hypothetical protein, conserved | 2,38 |
| LmjF23.0070 | agmatinase-like protein | 2,00 |
| LmjF23.0080 | hypothetical protein, conserved | 1,91 |
| **LmjF23.0090** | **hypothetical protein, conserved** | **2,73** |
| LmjF23.0100 | hypothetical protein, unknown function | 2,40 |
| LmjF23.0110 | mannose-1-phosphate guanyltransferase | 2,45 |
| LmjF23.0120 | hypothetical protein, conserved | 2,15 |
| LmjF23.0125 | cyclophilin type peptidyl-prolyl cis-trans isomerase, putative | 2,78 |
| **LmjF23.0130** | **vacuolar type h+ ATPase subunit, putative** | **2,74** |
| LmjF23.0140 | hypothetical protein, conserved | 2,46 |
| LmjF23.0180 | hypothetical protein, conserved | 1,76 |
| LmjF23.0190 | hypothetical protein, conserved | 2,03 |
| **LmjF23.0200** | **endoribonuclease L-PSP (pb5), putative** | **5,57** |
| LmjF23.0220 | multidrug resistance protein, putative | 4,47 |
| **LmjF23.0230** | **hypothetical protein, conserved** | **4,09** |
| **LmjF23.0240** | **terbinafine resistance locus protein (yip1)** | **5,38** |
| **LmjF23.0250** | **multidrug resistance protein, putative** | **5,48** |
| LmjF23.0270 | pteridine reductase 1 | 2,23 |
| LmjF23.0280 | hypothetical protein, conserved | 1,92 |
| LmjF23.0300 | tryptophanyl-tRNA synthetase, putative | 2,40 |
| LmjF23.0310 | hypothetical protein, conserved | 1,95 |
| LmjF23.0320 | hypothetical protein, conserved | 1,95 |
| **LmjF23.0330** | **hypothetical protein, conserved** | **2,89** |
| **LmjF23.0340** | **(H+)-ATPase G subunit, putative** | **3,64** |
| **LmjF23.0370** | **hypothetical protein, conserved** | **3,79** |
| **LmjF23.0380** | **ABC transporter-like protein** | **4,44** |
| LmjF23.0390 | hypothetical protein, conserved | 2,34 |
| LmjF23.0410 | hypothetical protein, unknown function | 2,68 |
| LmjF23.0420 | hypothetical protein, unknown function | 1,98 |
| LmjF23.0430 | aldose 1-epimerase-like protein | 2,31 |
| LmjF23.0440 | hypothetical protein, conserved | 2,14 |
| LmjF23.0450 | permease-like protein | 3,07 |
| LmjF23.0460 | trypanothione synthetase, putative | 2,07 |
| LmjF23.0470 | hypothetical protein, conserved | 2,14 |
| LmjF23.0500 | hypothetical protein, conserved | 2,17 |
| **LmjF23.0510** | **hypothetical protein, conserved** | **2,35** |
| LmjF23.0530 | ribosomal RNA methyltransferase-like protein | 1,78 |
| **LmjF23.0540** | **acetyl-CoA synthetase, putative** | **3,43** |
| LmjF23.0543 | 5’-3’ exonuclease XRNC, putative | 2,45 |
| LmjF23.0547 | hypothetical protein, conserved | 2,07 |
| **LmjF23.0820** | **hypothetical protein, conserved** | **1,82** |
| LmjF23.1590 | oxidoreductase-like protein | 3,08 |
| LmjF24.0060 | hypothetical protein, conserved | 1,85 |
| LmjF24.0290 | hypothetical protein, conserved | 1,83 |
| LmjF24.0370 | aspartate aminotransferase, putative | 1,78 |
| **LmjF24.0480** | **hypothetical predicted Kelch-domain protein** | **2,38** |
| LmjF24.0670 | protein kinase, putative | 1,82 |
| **LmjF24.0720** | **mitochondrial translocase subunit, putative** | **1,72** |
| **LmjF24.0760** | **DNA repair and recombination protein RAD54, putative** | **2,05** |
| LmjF24.1040 | hypothetical protein, unknown function | 2,14 |
| LmjF24.1270 | amastin-like surface protein-like protein | 2,13 |
| **LmjF24.1400** | **hypothetical protein, conserved** | **2,53** |
| LmjF24.1630 | succinate dehydrogenase flavoprotein, putative | 1,80 |
| LmjF24.1720 | cell division cycle protein, putative | 2,40 |
| LmjF24.1760 | hypothetical protein, conserved | 1,83 |
| **LmjF24.1910** | **hypothetical protein, conserved** | **3,14** |
| LmjF24.1950 | hypothetical protein, conserved | 2,75 |
| **LmjF25.0920** | **hypothetical protein, conserved** | **2,16** |
| LmjF25.0960 | gamma-tubulin | 2,18 |
| LmjF25.1320 | serine/threonine protein phosphatase, putative | 1,74 |
| LmjF25.1400 | hypothetical protein, conserved | 1,85 |
| LmjF25.1460 | hypothetical protein, conserved | 1,74 |
| **LmjF25.1470** | **cyclin** | **2,57** |
| LmjF25.2220 | hypothetical protein, conserved | 1,80 |
| LmjF26.0890 | 40S ribosomal protein S16, putative | 1,90 |
| **LmjF26.0970** | **hypothetical protein, conserved** | **2,35** |
| **LmjF26.1190** | **hypothetical protein, conserved** | **2,44** |
| LmjF26.1300 | hypothetical protein, conserved | 1,80 |
| LmjF26.1340 | DNA ligase k alpha, putative | 1,93 |
| LmjF26.1610 | proline oxidase, mitochondrial precursor-like protein | 1,74 |
| LmjF26.1640 | 40S ribosomal protein S33, putative | 1,88 |
| LmjF26.1710 | cytochrome c oxidase subunit V, putative | 1,78 |
| **LmjF26.1910** | **hypothetical protein, conserved** | **2,65** |
| LmjF26.2280 | nitrilase, putative | 1,80 |
| LmjF26.2310 | hypothetical protein, conserved | 1,88 |
| LmjF26.2560 | hypothetical protein, conserved | 1,95 |
| **LmjF26.2630** | **hypothetical protein, conserved** | **2,26** |
| **LmjF27.0050** | **DEAD-box helicase-like protein** | **1,84** |
| **LmjF27.0130** | **hypothetical protein, conserved** | **2,20** |
| **LmjF27.0210** | **hypothetical protein, conserved** | **1,94** |
| LmjF27.0220 | hypothetical protein, conserved | 1,82 |
| **LmjF27.0350** | **hypothetical protein, conserved** | **2,31** |
| LmjF27.0390 | hypothetical protein, conserved | 1,96 |
| **LmjF27.0430** | **hypothetical protein, conserved** | **2,61** |
| LmjF27.0470 | ABC transporter, putative | 1,88 |
| **LmjF27.0700** | **hypothetical protein, conserved** | **2,73** |
| LmjF27.0745 | hypothetical protein, conserved (pseudogene) | 1,78 |
| **LmjF27.0760** | **small GTP-binding protein Rab1, putative** | **2,46** |
| **LmjF27.0870** | **hypothetical protein, conserved** | **1,88** |
| **LmjF27.0980** | **ABC transporter, putative** | **2,05** |
| **LmjF27.1050** | **vesicular transport protein (CDC48 homologue), putative** | **1,73** |
| **LmjF27.1080** | **hypothetical protein, conserved** | **2,82** |
| **LmjF27.1120** | **hypothetical protein, conserved** | **2,01** |
| **LmjF27.1160** | **hypothetical protein, conserved** | **2,03** |
| LmjF27.1210 | translation initiation factor eIF2B subunit-like protein, putative | 1,80 |
| **LmjF27.1250** | **hypothetical protein, conserved** | **1,84** |
| **LmjF27.1265** | **cation transporter, putative** | **2,56** |
| **LmjF27.1300** | **hypothetical protein, conserved** | **2,21** |
| LmjF27.1420 | choline/ethanolamine kinase, putative | 1,69 |
| **LmjF27.1450** | **hypothetical protein, conserved** | **1,76** |
| **LmjF27.1460** | **proteasome regulatory non-ATP-ase subunit 3, putative** | **2,09** |
| **LmjF27.1580** | **amino acid transporter, putative** | **2,29** |
| LmjF27.1640 | hypothetical protein, conserved | 1,72 |
| **LmjF27.1680** | **hypothetical protein, conserved** | **1,69** |
| **LmjF27.1810** | **glycosomal phosphoenolpyruvate carboxykinase, putative** | **2,60** |
| **LmjF27.1870** | **trypanothione synthetase, putative** | **1,70** |
| **LmjF27.1980** | **FtsJ cell division protein, putative** | **1,86** |
| **LmjF27.2010** | **hypothetical protein, conserved** | **2,04** |
| LmjF27.2050 | ribonucleoside-diphosphate reductase small chain, putative | 1,85 |
| LmjF27.2110 | hypothetical protein, unknown function | 1,74 |
| **LmjF27.2270** | **hypothetical protein, conserved** | **2,51** |
| **LmjF27.2300** | **transcription elongation regulator-like protein** | **1,83** |
| **LmjF27.2320** | **protein phosphatase-like protein** | **2,37** |
| **LmjF27.2350** | **vesicle-associated membrane protein (VAMP), putative** | **1,77** |
| **LmjF28.0040** | **hypothetical protein, conserved** | **1,82** |
| LmjF28.0210 | histone H2B variant | 2,10 |
| LmjF28.0410 | hypothetical protein, conserved | 1,80 |
| **LmjF28.0550** | **RAD51 protein, putative** | **1,73** |
| **LmjF28.0890** | **ribonucleoside-diphosphate reductase large chain, putative** | **3,14** |
| LmjF28.1030 | ribosomal protein s20, putative | 1,71 |
| **LmjF28.1060** | **hypothetical protein, conserved** | **2,49** |
| LmjF28.1150 | long-chain-fatty-acid-coA ligase protein,putative | 2,10 |
| **LmjF28.1260** | **hypothetical protein, conserved** | **3,14** |
| LmjF28.1280 | putative phenylalanine-4-hydroxylase, putative | 1,79 |
| **LmjF28.1310** | **ATP-dependent RNA helicase, putative** | **2,20** |
| LmjF28.1380 | haloacid dehalogenase-like hydrolase-like protein | 2,38 |
| **LmjF28.1740** | **hypothetical protein, conserved** | **1,89** |
| LmjF28.1800 | hypothetical protein, conserved | 1,69 |
| **LmjF28.1810** | **hypothetical protein, conserved** | **2,12** |
| **LmjF28.1820** | **replication factor A, 51kDa subunit, putative** | **2,08** |
| **LmjF28.1940** | **hypothetical protein, conserved** | **1,85** |
| **LmjF28.2060** | **DNA-directed RNA polymerase-like protein** | **1,82** |
| LmjF28.2385 | DNA replication licensing factor, putative | 2,24 |
| LmjF28.2750 | activated protein kinase c receptor (LACK) | 2,36 |
| LmjF28.2960 | hypothetical protein, conserved | 2,05 |
| LmjF29.0120 | proteasome regulatory non-ATPase subunit, putative | 1,95 |
| LmjF29.0150 | hypothetical protein, conserved | 1,79 |
| **LmjF29.0250** | **oxidase-like protein** | **2,32** |
| LmjF29.0280 | D-lactate dehydrogenase-like protein | 1,86 |
| **LmjF29.0830** | **hypothetical protein, conserved** | **2,12** |
| LmjF29.0840 | hypothetical protein, conserved | 1,91 |
| **LmjF29.0850** | **high mobility group protein homolog tdp-1, putative** | **2,21** |
| **LmjF29.0865** | **hypothetical protein, conserved** | **3,31** |
| **LmjF29.0890** | **hypothetical protein, conserved** | **1,82** |
| LmjF29.0940 | hypothetical protein, conserved | 1,83 |
| **LmjF29.1260** | **hypothetical protein, conserved** | **1,74** |
| LmjF29.1270 | ATP-dependent Clp protease subunit, heat shock protein 100 (HSP100), putative | 1,88 |
| LmjF29.1280 | lipase domain protein, putative | 2,72 |
| LmjF29.2370 | 60S ribosomal protein L39, putative | 1,95 |
| **LmjF29.2510** | **6-phospho-1-fructokinase, putative** | **1,90** |
| LmjF30.0550 | hypothetical protein, conserved | 1,74 |
| **LmjF30.0620** | **ribosome biogenesis regulatory protein (RRS1), putative** | **1,86** |
| **LmjF30.1030** | **DnaJ-like protein** | **3,79** |
| LmjF30.1250 | pyridoxal kinase, putative | 2,05 |
| **LmjF30.1290** | **U3 small nuclear ribonucloprotein (snRNP), putative** | **2,01** |
| LmjF30.1370 | hypothetical protein, conserved | 1,95 |
| **LmjF30.1730** | **hypothetical protein, conserved** | **2,42** |
| LmjF30.1890 | hypothetical protein, conserved | 1,99 |
| LmjF30.2020 | hypothetical protein, unknown function | 2,33 |
| LmjF30.2110 | hypothetical protein, conserved | 1,76 |
| **LmjF30.2200** | **hypothetical protein, conserved** | **2,29** |
| LmjF30.2230 | hypothetical protein, unknown function | 1,86 |
| **LmjF30.2580** | **reticulon domain protein, 22 kDa potentially aggravating protein (paple22)** | **2,50** |
| LmjF30.2610 | RNA-binding protein, putative | 3,05 |
| LmjF30.2720 | hypothetical protein, conserved | 1,84 |
| LmjF30.2740 | TPR domain protein, conserved | 1,87 |
| **LmjF30.2900** | **aldehyde dehydrogenase, putative** | **1,86** |
| LmjF30.2950 | phospholipase c-like protein | 1,90 |
| LmjF30.3010 | hypothetical protein, conserved | 1,83 |
| LmjF30.3040 | eukaryotic translation initiation factor 3 subunit 7-like protein | 1,77 |
| **LmjF30.3140** | **hypothetical protein, conserved** | **1,80** |
| LmjF30.3150 | hypothetical protein, conserved | 1,95 |
| LmjF30.3190 | 3-hydroxy-3-methylglutaryl-CoA reductase, putative | 1,69 |
| LmjF30.3250 | ATP-dependent RNA helicase, putative | 1,74 |
| **LmjF30.3440** | **DNA ligase I, putative** | **2,68** |
| **LmjF30.3550** | **cytochrome p450-like protein** | **2,75** |
| LmjF30.3590 | 40S ribosomal protein S14 | 1,85 |
| LmjF31.0070 | helicase-like protein | 1,88 |
| **LmjF31.0080** | **hypothetical protein, conserved** | **2,52** |
| LmjF31.0230 | hypothetical protein, conserved | 1,84 |
| **LmjF31.0340** | **amino acid transporter aATP11, putative** | **1,69** |
| LmjF31.0450 | amastin, putative | 5,12 |
| **LmjF31.0470** | **hypothetical protein, conserved** | **2,54** |
| **LmjF31.0540** | **hypothetical protein, conserved** | **2,07** |
| **LmjF31.0570** | **amino acid transporter aATP11, putative** | **2,43** |
| **LmjF31.0740** | **hypothetical protein, conserved** | **3,67** |
| **LmjF31.0800** | **hypothetical protein, conserved** | **3,18** |
| LmjF31.0980 | hypothetical protein, conserved | 1,88 |
| **LmjF31.1010** | **hypothetical protein, conserved** | **1,95** |
| LmjF31.1030 | hypothetical protein, conserved | 1,76 |
| **LmjF31.1220** | **vacuolar-type proton translocating pyrophosphatase 1, putative** | **2,36** |
| LmjF31.1280 | p-glycoprotein e, putative | 1,79 |
| LmjF31.1290 | multidrug resistance protein, copy 1-like protein | 2,16 |
| LmjF31.1570 | cytochrome c oxidase VIII (COX VIII), putative | 2,07 |
| LmjF31.1600 | hypothetical protein, unknown function | 2,35 |
| **LmjF31.1610** | **diphthine synthase-like protein** | **2,59** |
| LmjF31.1950 | hypothetical protein, conserved | 1,74 |
| LmjF31.1980 | hypothetical protein, unknown function | 1,97 |
| LmjF31.2010 | hypothetical protein, unknown function | 2,01 |
| LmjF31.2060 | hypothetical protein, conserved | 2,20 |
| LmjF31.2080 | hypothetical protein, conserved | 1,94 |
| **LmjF31.2090** | **hypothetical protein, unknown function** | **2,28** |
| **LmjF31.2270** | **hypothetical protein, conserved** | **2,09** |
| **LmjF31.2310** | **3'-nucleotidase/nuclease precursor, putative** | **2,43** |
| LmjF31.2430 | hypothetical protein, unknown function | 1,92 |
| **LmjF31.2450** | **hypothetical protein, unknown function** | **3,43** |
| LmjF31.2550 | hypothetical protein, unknown function | 1,92 |
| LmjF31.2580 | ubiquinol-cytochrome-c reductase-like protein | 1,73 |
| **LmjF31.2600** | **calreticulin, putative** | **1,86** |
| LmjF31.2700 | hypothetical protein, conserved | 1,69 |
| **LmjF31.2760** | **kinetoplast-associated protein-like protein** | **2,44** |
| **LmjF31.2785** | **hypothetical protein, conserved** | **2,94** |
| LmjF31.2790 | adp-ribosylation factor, putative | 2,02 |
| **LmjF31.2860** | **protein kinase, putative** | **2,51** |
| LmjF31.2910 | hypothetical protein, conserved | 1,75 |
| **LmjF31.3130** | **methylcrotonoyl-coa carboxylase biotinylated subunitprotein-like protein** | **1,86** |
| LmjF31.3170 | hypothetical protein, unknown function | 1,72 |
| **LmjF32.0240** | **hypothetical protein, conserved** | **1,70** |
| **LmjF32.0450** | **40S ribosomal protein S2** | **1,92** |
| **LmjF32.0510** | **hypothetical protein, conserved** | **1,70** |
| **LmjF32.0700** | **ubiquitin carrier protein 4, putative** | **2,03** |
| LmjF32.0840 | hypothetical protein, conserved | 1,93 |
| **LmjF32.1600** | **hypothetical protein, conserved** | **1,86** |
| **LmjF32.1700** | **hypothetical protein, conserved** | **1,94** |
| **LmjF32.2150** | **hypothetical protein, conserved** | **1,69** |
| **LmjF32.2155** | **hypothetical protein, conserved** | **1,73** |
| LmjF32.2430 | hypothetical protein, conserved | 1,83 |
| **LmjF32.2520** | **hypothetical protein, unknown function** | **2,10** |
| **LmjF32.2640** | **cystathionine beta-lyase, putative** | **2,84** |
| **LmjF32.2930** | **tubulin-tyrosine ligase-like protein** | **2,25** |
| **LmjF32.2940** | **hypothetical protein, conserved** | **2,13** |
| LmjF33.0700 | hypothetical protein, conserved | 1,78 |
| **LmjF33.0920** | **40S ribosomal protein S3, putative** | **2,27** |
| **LmjF33.1140** | **hypothetical protein, conserved** | **6,33** |
| **LmjF33.1350** | **hypothetical protein, conserved** | **2,29** |
| LmjF33.1380 | mitogen activated protein kinase, putative | 1,73 |
| **LmjF33.1450** | **hypothetical protein, conserved** | **2,82** |
| LmjF33.1500 | hypothetical protein, conserved | 1,82 |
| **LmjF33.1590** | **hypothetical protein, conserved** | **3,04** |
| **LmjF33.1930** | **phosphoribosylpyrophosphate synthetase, putative** | **2,55** |
| LmjF33.2160 | hypothetical protein, conserved | 1,77 |
| LmjF33.2190 | hypothetical protein, unknown function | 1,72 |
| **LmjF33.3160** | **hypothetical protein, conserved** | **4,04** |
| LmjF33.3230 | 60S ribosomal protein L44, putative | 2,48 |
| **LmjF33.3240** | **h1 histone-like protein** | **2,09** |
| **LmjF34.0010** | **hypothetical protein, conserved** | **2,36** |
| **LmjF34.0070** | **ascorbate-dependent peroxidase, putative** | **2,51** |
| LmjF34.0370 | hypothetical protein, conserved | 2,34 |
| **LmjF34.0390** | **hypothetical protein, unknown function** | **1,99** |
| **LmjF34.0510** | **phosphoglycan beta 1,2 arabinosyltransferase** | **2,13** |
| LmjF34.0670 | ABC transporter-like protein | 1,71 |
| LmjF34.0860 | 60S ribosomal protein L13a, putative | 2,48 |
| LmjF34.1080 | amastin-like surface protein, putative | 2,48 |
| LmjF34.1320 | hypothetical protein, conserved | 1,80 |
| LmjF34.2040 | hypothetical protein, unknown function | 2,02 |
| **LmjF34.2050** | **ATP-dependent RNA helicase, putative** | **2,12** |
| LmjF34.3530 | hypothetical protein, conserved | 1,79 |
| LmjF34.3650 | 60S ribosomal protein L21, putative | 2,49 |
| LmjF34.3850 | aquaporin 9, putative | 1,96 |
| **LmjF34.4610** | **hypothetical protein, conserved** | **1,91** |
| LmjF35.0230 | hypothetical protein, conserved | 1,76 |
| LmjF35.0240 | 60S ribosomal protein L30 | 2,09 |
| LmjF35.0380 | hypothetical protein, conserved | 1,70 |
| **LmjF35.0880** | **hypothetical protein, conserved** | **2,89** |
| LmjF35.0950 | hypothetical protein, conserved | 1,70 |
| LmjF35.1020 | hypothetical protein, unknown function | 2,04 |
| **LmjF35.1040** | **hypothetical protein, conserved** | **2,13** |
| LmjF35.1240 | hypothetical protein, conserved | 1,80 |
| **LmjF35.1440** | **60S ribosomal protein L2, putative** | **1,93** |
| LmjF35.1650 | hypothetical protein, conserved | 1,83 |
| LmjF35.1680 | hypothetical protein, conserved | 1,73 |
| **LmjF35.1700** | **RNA 3'-terminal phosphate cyclase, putative** | **2,11** |
| LmjF35.1790 | DNA polymerase epsilon subunit B, putative | 1,75 |
| LmjF35.1920 | 60S ribosomal protein L36, putative | 2,14 |
| LmjF35.2050 | 60S ribosomal protein L32 | 1,72 |
| **LmjF35.2080** | **calcium motive p-type ATPase, putative** | **1,76** |
| LmjF35.2090 | kinesin, putative | 1,69 |
| LmjF35.2150 | hypothetical protein, conserved | 1,85 |
| LmjF35.2190 | 60S ribosomal protein L12, putative | 2,67 |
| **LmjF35.2750** | **hypothetical protein, conserved** | **3,78** |
| LmjF35.3230 | cystathione gamma lyase, putative | 1,91 |
| LmjF35.3440 | hypothetical protein, conserved | 1,91 |
| LmjF35.3510 | structural maintenance of chromosome (SMC) family protein, putative | 1,73 |
| **LmjF35.3680** | **ribulose-phosphate 3-epimerase, putative** | **2,70** |
| LmjF35.3750 | hypothetical protein, conserved | 1,74 |
| **LmjF35.4470** | **hypothetical protein, conserved** | **2,48** |
| LmjF35.4550 | hypothetical protein, conserved | 1,94 |
| **LmjF35.4700** | **MCAK-like kinesin, putative** | **2,48** |
| **LmjF35.4760** | **hypothetical protein, conserved** | **2,45** |
| **LmjF35.5040** | **polyadenylate-binding protein 1, putative** | **3,13** |
| **LmjF35.5100** | **60S ribosomal protein L37** | **2,39** |
| **LmjF35.5200** | **hypothetical protein, conserved** | **4,83** |
| LmjF36.0190 | elongation factor 2 | 1,86 |
| LmjF36.0370 | phosphatidylinositol-4-phosphate 5-kinase-like protein | 1,75 |
| LmjF36.0480 | hypothetical protein, conserved | 1,94 |
| **LmjF36.0550** | **cell division related protein kinase 2** | **2,41** |
| **LmjF36.0600** | **ubiquitin/ribosomal protein S27a, putative** | **1,90** |
| **LmjF36.0660** | **hypothetical protein, conserved** | **2,00** |
| LmjF36.0990 | 40S ribosomal protein S10, putative | 2,37 |
| **LmjF36.1160** | **hypothetical protein, conserved** | **1,78** |
| **LmjF36.1170** | **hypothetical protein, conserved** | **1,91** |
| LmjF36.1550 | hypothetical protein, conserved | 1,77 |
| **LmjF36.2000** | **hypothetical protein, conserved** | **3,48** |
| **LmjF36.2035** | **hypothetical protein, conserved** | **2,97** |
| LmjF36.2240 | hypothetical protein, conserved | 1,77 |
| **LmjF36.2360** | **tyrosine aminotransferase, putative** | **1,78** |
| **LmjF36.2590** | **membrane-bound acid phosphatase 2, putative** | **1,92** |
| LmjF36.2800 | hypothetical protein, conserved | 1,84 |
| LmjF36.2870 | 40S ribosomal protein S24e | 2,64 |
| **LmjF36.3070** | **fibrillarin** | **2,03** |
| LmjF36.3080 | lipoate protein ligase, putative | 2,11 |
| **LmjF36.3140** | **hypothetical protein, conserved** | **1,80** |
| LmjF36.3600 | hypothetical protein, conserved | 1,77 |
| **LmjF36.3760** | **60S ribosomal protein L10a, putative** | **1,85** |
| **LmjF36.3910** | **S-adenosylhomocysteine hydrolase** | **2,56** |
| LmjF36.3930 | telomerase reverse transcriptase, putative | 1,73 |
| LmjF36.4250 | protein kinase-like protein | 2,14 |
| LmjF36.4400 | ATP-dependent RNA helicase-like protein | 1,90 |
| LmjF36.4420 | 60S ribosomal protein L22, putative | 1,87 |
| LmjF36.4460 | hypothetical protein, conserved | 2,07 |
| LmjF36.4560 | hypothetical protein, conserved | 1,71 |
| **LmjF36.4580** | **hypothetical protein, conserved** | **2,12** |
| LmjF36.4990 | delta tubulin, putative | 1,79 |
| **LmjF36.5365** | **hypothetical protein, conserved** | **2,81** |
| **LmjF36.5820** | **mkiaa0324 protein-like protein** | **2,83** |
| **LmjF36.5845** | **kinetoplast-associated protein, putative** | **2,77** |
| LmjF36.5960 | glycerophosphoryl diester phosphodiesterase, putative | 2,44 |
| LmjF36.6780 | nuclear lim interactor-interacting factor, putative | 1,93 |
| LmjF36.6844 | hypothetical protein | 1,84 |
| **LmjF36.7000** | **hypothetical protein, conserved** | **4,20** |

GeneDB systematic IDs are related to *L. major* according to microarray labeled probes.

In **Bold,** genes up or downregulated in both Sb^III^2700.2 and Sb^III^2700.3 mutants.
