# Supplementary material for: Gene Expression Profiling and Molecular Characterization of Antimony Resistance in Leishmania amazonensis
Source: PLoS Negl Trop Dis. 2011 May 24;5(5):e1167. doi: 10.1371/journal.pntd.0001167 (PMC3101167; doi:10.1371/journal.pntd.0001167)
Supplement: Table S3 — Genes significantly modulated in antimony-resistant Leishmania amazonensis Ba199SbIII2700.3. The data were obtained by full genome microarray hybridization of Ba199SbIII2700.3 against Ba199 WT. (DOC) [file pntd.0001167.s003.doc]

**Table S3**. Genes significantly modulated in antimony-resistant *Leishmania amazonensis* Ba199Sb^III^2700.3. The data were obtained by full genome microarray hybridization of Ba199Sb^III^2700.3 against Ba199WT.

| **Gene Systematic ID** | **Gene description** | **Fold difference** |
| --- | --- | --- |
| **Downregulated Genes** | | |
| **LmjF01.0010** | **hypothetical protein, unknown function** | **-1,72** |
| **LmjF01.0260** | **hypothetical protein, conserved** | **-3,49** |
| LmjF01.0380 | hypothetical protein, conserved | -1,91 |
| LmjF01.0550 | hypothetical protein, conserved | -1,72 |
| **LmjF02.0040** | **aminopeptidase P1, putative** | **-1,93** |
| **LmjF02.0300** | **ABC1 transporter, putative** | **-2,36** |
| **LmjF02.0470** | **hypothetical protein, unknown function** | **-2,59** |
| **LmjF02.0590** | **hypothetical protein, conserved** | **-3,62** |
| LmjF03.0140 | hypothetical protein, conserved | -1,99 |
| **LmjF03.0470** | **hypothetical protein, conserved** | **-2,85** |
| **LmjF03.0530** | **hypothetical protein, conserved** | **-2,19** |
| LmjF03.0560 | hypothetical protein | -2,01 |
| **LmjF03.0900** | **peter pan protein, putative** | **-2,43** |
| **LmjF04.0110** | **hypothetical protein, conserved** | **-2,05** |
| **LmjF04.0170** | **hypothetical protein, conserved in leishmania** | **-1,88** |
| **LmjF04.0180** | **surface antigen-like protein** | **-1,99** |
| **LmjF04.0190** | **surface antigen-like protein** | **-2,42** |
| **LmjF04.0200** | **surface antigen-like protein** | **-2,34** |
| **LmjF04.0210** | **surface antigen-like protein** | **-2,10** |
| **LmjF04.0280** | **adenosine monophosphate deaminase, putative** | **-1,81** |
| **LmjF04.0440** | **serine/threonine protein kinase-like protein** | **-2,00** |
| **LmjF04.0450** | **calpain-like cysteine peptidase, putative** | **-1,73** |
| **LmjF04.0640** | **hypothetical protein** | **-2,90** |
| **LmjF04.0690** | **hypothetical protein** | **-2,01** |
| **LmjF04.0800** | **hypothetical protein, conserved** | **-1,80** |
| **LmjF04.0830** | **hypothetical protein, conserved** | **-1,96** |
| LmjF04.0890 | hypothetical protein, conserved | -1,99 |
| **LmjF04.1020** | **hypothetical protein, conserved** | **-1,75** |
| **LmjF04.1030** | **hypothetical protein, conserved** | **-2,43** |
| **LmjF04.1110** | **proton motive ATPase, putative** | **-2,53** |
| **LmjF04.1200** | **hypothetical protein, conserved** | **-1,69** |
| **LmjF04.1230** | **actin** | **-2,34** |
| **LmjF05.0420** | **dynein-light chain-protein, putative** | **-2,75** |
| LmjF05.0670 | hypothetical protein, conserved | -1,88 |
| LmjF06.0120 | cyclophilin | -1,85 |
| **LmjF06.0130** | **hypothetical protein, conserved** | **-3,46** |
| **LmjF06.0150** | **hypothetical protein, conserved** | **-2,04** |
| **LmjF06.0450** | **hypothetical protein, unknown function** | **-2,68** |
| **LmjF06.0750** | **hypothetical protein, conserved** | **-3,52** |
| **LmjF06.1050** | **protein disulfide isomerase** | **-2,22** |
| LmjF06.1070 | deoxyribose-phosphate aldolase, putative | -1,75 |
| **LmjF06.1080** | **hypothetical protein, unknown function** | **-2,38** |
| **LmjF06.1150** | **hypothetical protein, conserved** | **-1,81** |
| **LmjF06.1190** | **hypothetical protein, conserved** | **-2,20** |
| **LmjF07.0370** | **hypothetical protein, unknown function** | **-2,01** |
| LmjF07.0410 | hypothetical protein, conserved | -1,79 |
| **LmjF07.0420** | **hypothetical protein, conserved** | **-2,33** |
| **LmjF07.0890** | **hypothetical protein, unknown function** | **-1,98** |
| **LmjF07.1055** | **hypothetical protein, conserved** | **-2,14** |
| LmjF07.1110 | hypothetical protein, conserved | -1,80 |
| LmjF07.1130 | hypothetical protein, conserved | -1,80 |
| **LmjF08.0140** | **DNA repair protein, putative** | **-2,25** |
| **LmjF08.0460** | **hypothetical protein, unknown function** | **-2,04** |
| **LmjF08.0570** | **hypothetical protein, conserved** | **-1,76** |
| LmjF08.0600 | hypothetical protein, unknown function | -1,81 |
| LmjF08.1010 | cathepsin L-like protease | -1,86 |
| LmjF08.1080 | cathepsin L-like protease | -2,32 |
| LmjF08.1120 | hypothetical protein, conserved | -2,06 |
| LmjF08.1160 | hypothetical protein, conserved | -1,68 |
| LmjF08.1200 | hypothetical protein, conserved | -1,78 |
| LmjF08.1260 | hypothetical protein, conserved | -1,93 |
| **LmjF09.0300** | **hypothetical protein, conserved** | **-3,08** |
| **LmjF09.0420** | **tyrosine phosphatase, putative** | **-2,00** |
| **LmjF09.0530** | **leucine-rich repeat protein, putative** | **-2,69** |
| **LmjF09.0610** | **hypothetical protein, conserved** | **-2,08** |
| LmjF09.0760 | hypothetical protein, conserved | -1,72 |
| LmjF09.1380 | hypothetical protein, conserved | -1,96 |
| **LmjF09.1520** | **hypothetical protein, conserved** | **-1,77** |
| LmjF10.0185 | hypothetical protein | -1,79 |
| **LmjF10.0230** | **hypothetical protein, conserved** | **-2,36** |
| LmjF10.0260 | hypothetical protein, conserved | -1,77 |
| **LmjF10.0370** | **pteridine transporter ft4, putative** | **-1,76** |
| **LmjF10.0385** | **pteridine transporter ft3, putative** | **-2,26** |
| **LmjF10.0390** | **pteridine transporter ft3, putative** | **-2,60** |
| LmjF10.0650 | hypothetical protein, unknown function | -1,96 |
| LmjF10.0730 | deaminase, putative | -1,78 |
| LmjF10.1080 | hypothetical protein, conserved | -1,86 |
| **LmjF10.1140** | **hypothetical protein, conserved** | **-3,03** |
| **LmjF11.0160** | **hypothetical protein, conserved** | **-1,88** |
| LmjF11.0300 | hypothetical protein, conserved | -1,75 |
| **LmjF11.0320** | **PIF1 helicase-like protein, putative** | **-3,28** |
| LmjF11.0990 | adaptin-related protein-like protein | -1,70 |
| **LmjF11.1240** | **ABC1 transporter, putative** | **-1,99** |
| **LmjF11.1270** | **ABC transporter, putative** | **-2,15** |
| **LmjF11.1290** | **ABC transporter, putative** | **-2,84** |
| **LmjF11.1310** | **hypothetical protein, conserved** | **-2,03** |
| **LmjF12.0150** | **leucine rich repeat protein, putative** | **-2,56** |
| **LmjF12.1015** | **hypothetical protein, unknown function** | **-1,96** |
| **LmjF12.1040** | **surface antigen protein, putative** | **-2,10** |
| **LmjF12.1060** | **surface antigen protein, putative** | **-1,94** |
| **LmjF12.1070** | **surface antigen protein 2, putative** | **-2,07** |
| **LmjF12.1290** | **hypothetical protein, conserved** | **-2,30** |
| **LmjF13.0190** | **hypothetical protein, unknown function** | **-1,79** |
| **LmjF13.0390** | **alpha tubulin** | **-2,55** |
| LmjF13.0950 | actin-like protein, putative | -1,83 |
| LmjF13.1310 | hypothetical protein, unknown function | -1,72 |
| LmjF13.1390 | hypothetical protein, conserved | -1,75 |
| **LmjF13.1530** | **phospholipid-transporting ATPase 1-like protein** | **-2,83** |
| LmjF13.1570 | phosphoprotein phosphatase-like protein | -2,02 |
| **LmjF14.0510** | **stearic acid desaturase, putative** | **-2,26** |
| **LmjF14.0700** | **fatty acid elongase, putative** | **-2,60** |
| **LmjF14.0720** | **fatty acid elongase, putative** | **-2,15** |
| **LmjF14.0870** | **hypothetical protein, conserved** | **-1,96** |
| LmjF14.0900 | protein phosphatase 2C-like protein | -1,77 |
| **LmjF14.0920** | **mitochondrial DNA polymerase I protein C, putative** | **-1,94** |
| LmjF14.0940 | hypothetical protein, conserved | -1,83 |
| LmjF14.1070 | protein kinase, putative | -1,84 |
| **LmjF14.1080** | **hypothetical protein, conserved** | **-3,15** |
| **LmjF14.1220** | **hypothetical protein, conserved** | **-1,75** |
| **LmjF14.1250** | **hypothetical protein, unknown function** | **-2,07** |
| **LmjF14.1390** | **hypothetical protein, conserved** | **-2,16** |
| LmjF14.1420 | hypothetical protein, conserved | -1,79 |
| **LmjF15.0110** | **hypothetical protein, conserved** | **-1,72** |
| **LmjF15.0350** | **hypothetical protein, conserved** | **-1,90** |
| LmjF15.0900 | hypothetical protein, conserved | -1,70 |
| **LmjF15.1150** | **developmentally regulated protein, putative** | **-1,97** |
| LmjF15.1345 | hypothetical protein, conserved | -1,75 |
| LmjF15.1410 | hypothetical protein, conserved | -1,75 |
| **LmjF15.1480** | **cAMP specific phosphodiesterase, putative** | **-1,83** |
| **LmjF15.1510** | **hypothetical protein, conserved** | **-2,65** |
| **LmjF16.0150** | **hypothetical protein, conserved** | **-1,98** |
| LmjF16.0150 | hypothetical protein, conserved | -1,92 |
| LmjF16.0610 | histone h3, putative | -1,93 |
| LmjF16.0610 | histone h3, putative | -1,91 |
| LmjF16.0900 | hypothetical protein, conserved | -1,72 |
| LmjF16.0940 | dynein light chain, putative | -1,76 |
| LmjF16.1030 | hypothetical protein, conserved | -1,80 |
| LmjF16.1040 | hypothetical protein, unknown function | -1,79 |
| **LmjF16.1250** | **hypothetical protein, conserved** | **-2,07** |
| LmjF16.1400 | trafficking protein particle complex subunit-like protein | -1,69 |
| LmjF16.1430 | paraflagellar rod protein 2C | -1,92 |
| LmjF16.1650 | hypothetical protein, conserved | -1,86 |
| **LmjF17.0170** | **hypothetical protein, conserved** | **-1,76** |
| **LmjF17.0237** | **receptor-type adenylate cyclase b** | **-2,04** |
| **LmjF18.0180** | **hypothetical protein, unknown function** | **-2,01** |
| **LmjF18.0270** | **protein kinase, putative** | **-2,98** |
| **LmjF18.0470** | **hypothetical protein, conserved** | **-2,23** |
| **LmjF18.0760** | **hypothetical protein, conserved** | **-1,75** |
| **LmjF18.1230** | **hypothetical protein, conserved** | **-2,07** |
| **LmjF19.0430** | **hypothetical protein, conserved** | **-1,97** |
| **LmjF19.0848** | **microtubial binding protein-like protein** | **-2,51** |
| **LmjF19.0850** | **microtubule associated protein-like protein** | **-1,87** |
| **LmjF19.0910** | **microtubule associated protein-like** | **-2,39** |
| **LmjF19.1100** | **hypothetical protein, conserved** | **-2,19** |
| LmjF19.1347 | hypothetical protein, conserved | -1,85 |
| **LmjF19.1360** | **hypothetical protein, unknown function** | **-3,03** |
| **LmjF20.0150** | **hypothetical protein** | **-1,74** |
| **LmjF20.0310** | **hypothetical protein, conserved** | **-1,77** |
| LmjF20.0360 | hypothetical protein, conserved | -1,75 |
| **LmjF20.0600** | **hypothetical protein, conserved** | **-1,77** |
| **LmjF20.1040** | **hypothetical protein, conserved** | **-1,73** |
| **LmjF20.1470** | **hypothetical protein, unknown function** | **-1,77** |
| LmjF21.0260 | hypothetical protein, conserved | -1,77 |
| LmjF21.0650 | hypothetical protein, conserved | -1,93 |
| **LmjF21.0875** | **hypothetical protein, conserved** | **-3,18** |
| LmjF21.0882 | hypothetical protein, conserved | -1,74 |
| **LmjF21.0960** | **hypothetical protein, conserved** | **-2,04** |
| **LmjF21.0970** | **hypothetical protein, conserved** | **-2,18** |
| LmjF21.1110 | hypothetical protein, conserved | -1,75 |
| **LmjF21.1520** | **hypothetical protein, unknown function** | **-3,12** |
| LmjF21.1650 | protein kinase, putative | -1,85 |
| **LmjF22.0040** | **hypothetical protein, conserved** | **-2,25** |
| **LmjF22.0250** | **hypothetical protein, unknown function** | **-1,76** |
| **LmjF22.0450** | **hypothetical protein, conserved** | **-2,49** |
| **LmjF22.1070** | **hypothetical protein, conserved** | **-2,09** |
| LmjF22.1300 | polyprotein, putative | -1,78 |
| **LmjF23.0125** | cyclophilin type peptidyl-prolyl cis-trans isomerase, putative | -2,24 |
| LmjF23.1070 | hydrophilic surface protein | -1,86 |
| **LmjF23.1088** | **hydrophilic surface protein 2** | **-2,13** |
| LmjF23.1170 | membrane-bound acid phosphatase 2 | -1,89 |
| **LmjF23.1550** | **hypothetical protein, conserved** | **-1,93** |
| **LmjF23.1660** | **metallo-beta-lactamase family protein-like protein** | **-2,24** |
| **LmjF24.0280** | **dynein intermediate-chain-like protein** | **-1,97** |
| **LmjF24.0340** | **hypothetical protein, conserved** | **-1,69** |
| **LmjF24.0400** | **hypothetical protein, conserved** | **-2,94** |
| LmjF24.0430 | hypothetical protein, unknown function | -2,02 |
| LmjF24.0770 | malic enzyme, putative | -1,82 |
| LmjF24.0940 | hypothetical protein, conserved | -1,71 |
| **LmjF24.1160** | **hypothetical protein, unknown function** | **-2,11** |
| LmjF24.1315 | peptidyl-prolyl cis-trans isomerase-like protein | -1,72 |
| **LmjF24.1560** | **hypothetical protein, conserved** | **-1,84** |
| **LmjF24.1600** | **hypothetical protein, unknown function** | **-2,12** |
| LmjF24.1610 | ring-box protein, putative | -1,98 |
| LmjF24.1880 | cyclin 11, putative | -1,69 |
| **LmjF24.1900** | **hypothetical protein, conserved** | **-1,90** |
| **LmjF24.2040** | **hypothetical protein, conserved** | **-2,75** |
| **LmjF25.0240** | **hypothetical protein, conserved** | **-1,74** |
| **LmjF25.0350** | **hypothetical protein, conserved** | **-2,24** |
| LmjF25.0900 | hypothetical protein, unknown function | -1,82 |
| **LmjF25.0950** | **hypothetical protein, conserved** | **-1,70** |
| **LmjF25.1670** | **rev7, putative** | **-1,98** |
| **LmjF25.1690** | **hypothetical protein, conserved** | **-2,55** |
| LmjF25.1705 | hypothetical protein, conserved | -1,82 |
| **LmjF25.1740** | **hypothetical protein, conserved** | **-2,12** |
| **LmjF25.1910** | **hypothetical protein, conserved** | **-2,49** |
| LmjF26.0040 | protein kinase, putative | -1,74 |
| LmjF26.0690 | hypothetical protein, conserved | -2,05 |
| **LmjF26.0780** | **hypothetical protein, conserved** | **-2,36** |
| LmjF26.0810 | glutathione peroxidase-like protein, putative | -1,74 |
| LmjF26.0870 | hypothetical protein, conserved | -1,80 |
| **LmjF26.0940** | **hypothetical protein, conserved** | **-1,71** |
| **LmjF26.1110** | **hypothetical protein, conserved** | **-1,95** |
| **LmjF26.1450** | **hypothetical protein, conserved** | **-1,77** |
| **LmjF26.1490** | **hypothetical protein, unknown function** | **-2,58** |
| **LmjF26.1500** | **hypothetical protein, unknown function** | **-1,93** |
| **LmjF26.1830** | **hypothetical protein, conserved** | **-3,18** |
| **LmjF26.1980** | **hypothetical protein, conserved** | **-2,12** |
| **LmjF26.2260** | **syntaxin binding protein 1, putative** | **-2,44** |
| LmjF26.2470 | hypothetical protein, conserved | -1,85 |
| **LmjF26.2550** | **hypothetical protein, conserved** | **-1,79** |
| **LmjF26.2570** | **protein kinase, putative** | **-2,35** |
| **LmjF26.2670** | **p-glycoprotein-like protein** | **-2,93** |
| **LmjF26.2680** | **hypothetical protein, unknown function** | **-10,15** |
| LmjF27.0380 | nucleoporin, putative | -1,70 |
| LmjF27.0860 | hypothetical protein, conserved | -2,04 |
| **LmjF27.1950** | **hypothetical protein, conserved** | **-1,75** |
| LmjF27.2430 | hypothetical protein, conserved | -1,89 |
| **LmjF27.2460** | **protein kinase, putative** | **-2,29** |
| **LmjF28.0220** | **hypothetical protein, conserved** | **-2,46** |
| LmjF28.0260 | hypothetical protein, conserved | -1,77 |
| **LmjF28.0370** | **hypothetical protein, unknown function** | **-2,72** |
| LmjF28.0950 | hypothetical protein, conserved | -1,78 |
| **LmjF28.1710** | **hypothetical protein, conserved** | **-1,68** |
| **LmjF28.2210** | **glycoprotein 96-92, putative** | **-1,72** |
| **LmjF28.2300** | **hypothetical protein, conserved** | **-2,00** |
| LmjF28.2450 | hypothetical protein, conserved | -1,71 |
| **LmjF28.2490** | **hypothetical protein, conserved** | **-1,91** |
| **LmjF28.2920** | **hypothetical protein, conserved** | **-1,96** |
| **LmjF28.2960** | **hypothetical protein, conserved** | **-2,35** |
| LmjF28.3005 | glucose 6-phosphate N-acetyltransferase, putative | -1,72 |
| **LmjF29.0090** | **GTP-binding protein-like protein** | **-1,84** |
| **LmjF29.0570** | **hypothetical protein, conserved** | **-2,24** |
| **LmjF29.1000** | **hypothetical protein, conserved** | **-1,78** |
| **LmjF29.1440** | **clathrin coat assembly protein AP19, putative** | **-1,74** |
| **LmjF29.1470** | **hypothetical protein, conserved** | **-2,48** |
| **LmjF29.1570** | **glutamamyl carboxypeptidase, putative** | **-1,97** |
| **LmjF29.1710** | **MutS-like protein** | **-2,16** |
| **LmjF29.1740** | **histone H2A, putative** | **-3,85** |
| **LmjF29.1920** | **hypothetical protein, unknown function** | **-2,01** |
| **LmjF29.2030** | **N-acetylglucosaminyl transferase component, putative** | **-2,35** |
| **LmjF29.2410** | **hypothetical protein, conserved** | **-2,42** |
| **LmjF29.2480** | **hypothetical protein, conserved** | **-2,15** |
| **LmjF29.2590** | **hypothetical protein, conserved** | **-1,86** |
| **LmjF29.2720** | **protein kinase, putative** | **-1,88** |
| **LmjF30.0840** | **hypothetical protein, conserved** | **-1,81** |
| **LmjF30.1050** | **mitochondrial carrier protein-like protein** | **-2,64** |
| **LmjF30.1170** | **hypothetical protein, unknown function** | **-1,85** |
| **LmjF30.1985** | **hypothetical protein, conserved** | **-2,38** |
| **LmjF30.2420** | **hypothetical protein, conserved** | **-1,93** |
| **LmjF30.2650** | **hypothetical protein, conserved** | **-1,73** |
| **LmjF30.2790** | **hypothetical protein, conserved** | **-1,94** |
| **LmjF30.2920** | **hypothetical protein, conserved** | **-3,34** |
| LmjF31.0100 | O-sialoglycoprotein endopeptidase, putative | -1,92 |
| LmjF31.0160 | DNA-directed RNA polymerase II subunit 2, putative | -1,73 |
| **LmjF31.0410** | **calpain-like cysteine peptidase, putative** | **-1,80** |
| LmjF31.0480 | hypothetical protein, conserved | -1,81 |
| LmjF31.0760 | hypothetical protein, unknown function | -1,84 |
| **LmjF31.0810** | **hypothetical protein, conserved** | **-2,83** |
| LmjF31.0940 | hypothetical protein | -1,83 |
| **LmjF31.1340** | **hypothetical protein, conserved** | **-2,55** |
| LmjF31.1410 | hypothetical protein, conserved | -1,79 |
| **LmjF31.1440** | **hypothetical protein, unknown function** | **-6,63** |
| **LmjF31.1760** | **hypothetical protein, unknown function** | **-2,11** |
| **LmjF31.1810** | **acetylornithine deacetylase-like protein** | **-1,80** |
| LmjF31.2050 | hypothetical protein, unknown function | -1,73 |
| LmjF31.2130 | hypothetical protein, conserved | -2,05 |
| **LmjF31.2320** | **helicase-like protein** | **-1,82** |
| **LmjF31.2400** | **hypothetical protein, unknown function** | **-2,83** |
| **LmjF32.0810** | **serine/threonine protein kinase, putative** | **-1,82** |
| **LmjF32.0930** | **hypothetical protein, conserved** | **-2,84** |
| **LmjF32.2210** | **hypothetical protein, conserved** | **-3,09** |
| **LmjF32.3070** | **hypothetical protein, conserved** | **-2,39** |
| LmjF32.3080 | ABC transporter, putative – ABCB3 | -2,53 |
| **LmjF32.3150** | **hypothetical protein, conserved** | **-2,19** |
| **LmjF32.3790** | **cyclin-dependent kinase regulatory subunit** | **-1,78** |
| **LmjF32.3960** | **hypothetical protein, conserved** | **-2,00** |
| **LmjF33.0160** | **hypothetical protein, unknown function** | **-1,69** |
| **LmjF33.0290** | **glucose transporter/membrane transporter D2, putative** | **-1,82** |
| **LmjF33.0500** | **hypothetical protein, conserved** | **-2,07** |
| **LmjF33.0610** | **hypothetical protein, conserved** | **-1,83** |
| LmjF33.0740 | hypothetical protein, conserved | -1,86 |
| **LmjF33.0820** | **beta tubulin** | **-2,61** |
| **LmjF33.0880** | **hypothetical protein, conserved** | **-2,57** |
| **LmjF33.0930** | **hypothetical protein, conserved** | **-2,29** |
| **LmjF33.1080** | **hypothetical protein, conserved** | **-1,79** |
| **LmjF33.1410** | **hypothetical protein, conserved** | **-2,24** |
| LmjF33.1540 | elongation factor 1-gamma, putative | -1,85 |
| LmjF33.1660 | hypothetical protein, conserved | -1,99 |
| **LmjF33.1830** | **protein kinase, putative** | **-1,98** |
| LmjF33.2850 | D-alanyl-glycyl endopeptidase-like protein | -1,84 |
| **LmjF33.2890** | **hypothetical protein, conserved** | **-2,36** |
| **LmjF33.2900** | **hypothetical protein, unknown function** | **-2,23** |
| LmjF33.2910 | hypothetical protein, conserved | -2,00 |
| **LmjF33.2930** | **hypothetical protein, conserved** | **-2,09** |
| **LmjF33.3000** | **hypothetical protein, unknown function** | **-1,71** |
| **LmjF34.0280** | **calpain-like cysteine peptidase, putative** | **-1,73** |
| **LmjF34.0410** | **hypothetical protein, conserved** | **-1,78** |
| **LmjF34.0460** | **hypothetical protein, unknown function** | **-2,96** |
| **LmjF34.0850** | **serine/threonine-protein phosphatase PP1, putative** | **-2,10** |
| **LmjF34.1350** | **hypothetical protein, conserved** | **-1,98** |
| **LmjF34.1380** | **hypothetical protein, unknown function** | **-2,46** |
| **LmjF34.1490** | **hypothetical protein, conserved** | **-2,76** |
| **LmjF34.1960** | **amastin-like surface protein, putative** | **-1,90** |
| **LmjF34.2030** | **hypothetical protein, conserved** | **-1,84** |
| **LmjF34.2190** | **dual specificity protein phosphatase, putative** | **-2,12** |
| LmjF34.2330 | clathrin coat assembly protein AP17, putative | -1,80 |
| **LmjF34.2370** | **hypothetical protein, conserved** | **-2,78** |
| LmjF34.2590 | adaptor complex subunit medium chain 3, putative | -1,70 |
| **LmjF34.2765** | **hypothetical protein, conserved** | **-2,81** |
| LmjF34.3130 | hypothetical protein, conserved | -1,79 |
| **LmjF34.3320** | **hypothetical protein, conserved** | **-1,92** |
| LmjF34.3660 | transmembrane/endomembrane-like protein | -2,07 |
| **LmjF34.3710** | **hypothetical protein, conserved** | **-1,83** |
| **LmjF34.3770** | **hypothetical protein, conserved** | **-2,02** |
| **LmjF34.4120** | **hypothetical protein, conserved** | **-2,60** |
| **LmjF34.4540** | **hypothetical protein, conserved** | **-1,69** |
| **LmjF34.4620** | **hypothetical protein, conserved** | **-1,70** |
| **LmjF35.0430** | **hypothetical protein, conserved** | **-1,93** |
| **LmjF35.0480** | **hypothetical protein, conserved** | **-1,75** |
| **LmjF35.0500** | **proteophosphoglycan ppg3, putative** | **-2,86** |
| **LmjF35.0540** | **proteophosphoglycan 5** | **-4,36** |
| **LmjF35.0550** | **proteophosphoglycan ppg1** | **-3,52** |
| **LmjF35.0740** | **hypothetical protein, conserved** | **-2,45** |
| LmjF35.1050 | protein kinase, putative | -1,72 |
| LmjF35.1150 | oligosaccharyl transferase-like protein | -1,78 |
| LmjF35.1190 | NADH-dependent fumarate reductase, putative | -1,91 |
| **LmjF35.1360** | **hypothetical protein, unknown function** | **-2,46** |
| **LmjF35.1620** | **hypothetical protein, conserved** | **-2,08** |
| **LmjF35.2790** | **hypothetical protein, conserved** | **-2,29** |
| LmjF35.3220 | hypothetical protein, conserved | -1,76 |
| **LmjF35.3300** | **hypothetical protein, unknown function** | **-2,03** |
| **LmjF35.3530** | **hypothetical protein, unknown function** | **-2,25** |
| **LmjF35.3550** | **hypothetical protein, conserved** | **-2,31** |
| LmjF35.3610 | peptidyl-prolyl cis-trans isomerase (cyclophilin), putative | -1,75 |
| **LmjF35.3660** | **hypothetical protein, conserved** | **-2,22** |
| **LmjF35.3900** | **hypothetical protein, conserved** | **-1,88** |
| **LmjF35.4045** | **hypothetical protein, conserved** | **-1,97** |
| **LmjF35.4170** | **hypothetical protein, conserved** | **-1,86** |
| **LmjF35.4230** | **hypothetical protein, conserved** | **-2,60** |
| **LmjF35.4610** | **hypothetical protein, conserved** | **-2,26** |
| LmjF35.5140 | hypothetical protein, unknown function | -1,80 |
| **LmjF35.5220** | **hypothetical protein, conserved** | **-1,82** |
| **LmjF36.0230** | **peptidyl-prolyl cis-trans isomerase, putative** | **-2,32** |
| **LmjF36.0280** | **hypothetical protein, conserved** | **-2,45** |
| LmjF36.0320 | proteasome beta 2 subunit, putative | -1,72 |
| **LmjF36.0420** | **amino acid permease-like protein** | **-2,48** |
| LmjF36.0790 | hypothetical protein, unknown function | -1,99 |
| LmjF36.1280 | tuzin-like protein | -1,78 |
| **LmjF36.1450** | **hypothetical protein, conserved** | **-2,60** |
| LmjF36.1670 | hypothetical protein, conserved | -1,85 |
| **LmjF36.1680** | **hypothetical protein, conserved** | **-1,83** |
| LmjF36.1690 | hypothetical protein, conserved | -2,10 |
| LmjF36.1730 | hypothetical protein, conserved | -1,83 |
| **LmjF36.2090** | **hypothetical protein, conserved** | **-2,39** |
| **LmjF36.2550** | **hypothetical protein, conserved** | **-2,20** |
| LmjF36.2730 | D-tyrosyl-tRNA deacylase, putative | -1,86 |
| **LmjF36.2890** | **ATP-binding cassette protein, putative** | **-2,96** |
| LmjF36.3230 | oxidoreductase, putative | -1,74 |
| **LmjF36.3620** | **hypothetical protein, conserved** | **-1,71** |
| **LmjF36.3920** | **hypothetical protein, conserved** | **-2,33** |
| **LmjF36.4130** | **hypothetical protein, unknown function** | **-2,04** |
| **LmjF36.4140** | **hypothetical protein, unknown function** | **-1,90** |
| **LmjF36.4145** | **transcription factor S-II-like protein** | **-2,30** |
| **LmjF36.4530** | **ABC1 protein, putative** | **-2,07** |
| LmjF36.4880 | hypothetical protein, conserved | -1,73 |
| **LmjF36.5210** | **hypothetical protein, conserved** | **-2,30** |
| **LmjF36.5340** | **hypothetical protein, conserved** | **-2,09** |
| **LmjF36.5630** | **hypothetical protein, conserved** | **-2,16** |
| **LmjF36.5870** | **hypothetical protein, conserved** | **-2,05** |
| **LmjF36.6110** | **centrin, putative** | **-1,78** |
| **LmjF36.6290** | **glucose transporter, lmgt2, putative** | **-19,87** |
| **LmjF36.6300** | **glucose transporter, putative** | **-17,15** |
| **LmjF36.6430** | **protein transport protein sec23-like protein** | **-2,28** |
| **LmjF36.6480** | **histidine secretory acid phosphatase, putative** | **-2,41** |
| **LmjF36.6530** | **hypothetical protein, conserved** | **-1,75** |
| **Upregulated Genes** | | |
| LmjF01.0090 | hypothetical protein, conserved | 1,69 |
| **LmjF01.0780** | **eukaryotic initiation factor 4a, putative** | **1,88** |
| **LmjF02.0200** | **phosphoglycan beta 1,3 galactosyltransferase-like protein** | **1,83** |
| **LmjF02.0460** | **hypothetical protein, conserved** | **2,25** |
| **LmjF02.0660** | **hypothetical protein, conserved** | **1,91** |
| **LmjF03.0030** | **D-3-phosphoglycerate dehydrogenase-like protein** | **1,87** |
| **LmjF03.0200** | **delta-1-pyrroline-5-carboxylate dehydrogenase, putative** | **1,78** |
| LmjF03.0230 | long chain fatty Acyl CoA synthetase, putative | 1,69 |
| **LmjF03.0310** | **hypothetical protein** | **2,19** |
| **LmjF03.0440** | **60S acidic ribosomal protein P2, putative** | **1,92** |
| **LmjF03.0460** | **hypothetical protein** | **2,40** |
| LmjF03.0580 | hypothetical protein, conserved | 1,69 |
| **LmjF03.0840** | **hypothetical protein, conserved** | **1,81** |
| LmjF03.0980 | elongation initiation factor 2 alpha subunit, putative | 2,34 |
| **LmjF04.0040** | **hypothetical protein** | **3,51** |
| LmjF05.0010 | hypothetical protein, conserved | 1,88 |
| **LmjF05.0080** | **hypothetical protein, conserved** | **1,81** |
| LmjF05.0090 | hypothetical protein, conserved | 1,86 |
| LmjF05.0120 | hypothetical protein, conserved | 1,92 |
| LmjF05.0200 | hypothetical protein, conserved | 1,98 |
| LmjF05.0310 | glutaredoxin-like protein | 1,71 |
| **LmjF05.0350** | **trypanothione reductase** | **2,32** |
| **LmjF05.0400** | **structural maintenance of chromosome (SMC), putative** | **2,52** |
| LmjF05.0450 | hypothetical protein, conserved | 2,06 |
| **LmjF05.0460** | **GTPase, putative** | **2,90** |
| LmjF05.0490 | hypothetical protein, conserved | 1,70 |
| LmjF05.0720 | phophatase-like protein | 1,81 |
| LmjF05.0740 | hypothetical protein, conserved | 1,95 |
| LmjF05.0800 | MYND zinc finger (ZnF) domain-like protein | 2,00 |
| LmjF05.0980 | NADH-ubiquinone oxidoreductase, mitochondrial, putative | 1,98 |
| LmjF05.0990 | hypothetical protein, conserved | 1,87 |
| **LmjF05.1000** | **hypothetical protein, conserved** | **2,10** |
| LmjF05.1050 | hypothetical protein, unknown function | 1,90 |
| LmjF05.1090 | double-strand-break repair protein rad21 homolog, putative | 2,04 |
| **LmjF05.1110** | **hypothetical protein, conserved** | **3,03** |
| LmjF05.1200 | prefoldin subunit, putative | 2,09 |
| LmjF05.1210 | protein phosphatase type 1 regulator-like protein | 1,77 |
| LmjF06.0350 | NAD(p)-dependent steroid dehydrogenase-like protein | 2,06 |
| **LmjF06.0560** | **deoxyuridine triphosphatase, putative** | **2,23** |
| **LmjF06.0610** | **carbonic anhydrase family protein, putative** | **1,93** |
| **LmjF06.0860** | **dihydrofolate reductase-thymidylate synthase** | **2,12** |
| **LmjF06.0930** | **2,4-dienoyl-coa reductase-like protein** | **1,75** |
| **LmjF07.0350** | **hypothetical protein, conserved** | **2,47** |
| **LmjF07.0470** | **hypothetical protein, conserved** | **1,86** |
| LmjF07.0610 | hypothetical protein, conserved | 1,69 |
| **LmjF07.0710** | **centrin, putative** | **1,68** |
| **LmjF07.0802** | **hypothetical protein, conserved** | **2,42** |
| **LmjF07.0860** | **hypothetical protein, unknown function** | **1,86** |
| **LmjF08.0170** | **hypothetical protein, unknown function** | **1,80** |
| LmjF09.0400 | protein kinase, putative | 1,81 |
| LmjF09.0600 | cyclin 1, putative | 1,94 |
| LmjF10.0219 | hypothetical protein, unknown function | 1,74 |
| **LmjF10.0220** | **hypothetical protein, unknown function** | **2,45** |
| **LmjF10.0860** | **hypothetical protein, conserved** | **1,71** |
| **LmjF10.1290** | **hypothetical protein, conserved** | **1,84** |
| LmjF11.0400 | tubulin-tyrsoine ligase-like protein | 1,74 |
| **LmjF11.0470** | **pumilio-repeat, RNA-binding protein, putative** | **2,46** |
| LmjF11.0480 | hypothetical protein, conserved | 1,79 |
| **LmjF11.0650** | **hypothetical protein, conserved** | **2,28** |
| LmjF11.0675 | hypothetical protein, conserved in leishmania | 2,75 |
| LmjF11.0675 | hypothetical protein, conserved in leishmania | 2,48 |
| LmjF11.0980 | hypothetical protein, conserved | 1,76 |
| LmjF12.0060 | ribonuclease mar1 | 2,76 |
| LmjF12.0100 | hypothetical protein, conserved | 1,75 |
| LmjF12.0130 | protein kinase, putative | 2,02 |
| LmjF12.0240 | hypothetical protein, unknown function | 2,22 |
| LmjF12.0250 | cysteinyl-tRNA synthetase, putative | 1,68 |
| **LmjF12.0310** | **hypothetical protein, conserved** | **2,08** |
| **LmjF12.0350** | **hypothetical protein, conserved** | **1,69** |
| LmjF12.0380 | hypothetical protein, unknown function | 1,92 |
| **LmjF12.0400** | **3'-nucleotidase/nuclease, putative** | **2,34** |
| LmjF12.0470 | hypothetical protein, unknown function | 1,77 |
| **LmjF12.0480** | **hypothetical protein, unknown function** | **1,77** |
| **LmjF12.0490** | **hypothetical protein, unknown function** | **2,07** |
| LmjF12.0530 | glucose-6-phosphate isomerase | 3,18 |
| **LmjF12.0670** | **cytochrome c oxidase subunit iv** | **2,09** |
| LmjF12.1240 | hypothetical protein, unknown function | 1,81 |
| LmjF12.1270 | arginine N-methyltransferase-like protein | 2,37 |
| LmjF12.1330 | serine peptidase, putative | 2,17 |
| **LmjF13.0450** | **hypothetical protein, conserved** | **2,43** |
| **LmjF13.1210** | **nucleobase/nucleoside transporter 8.1, putative** | **4,35** |
| **LmjF13.1620** | **squalene monooxygenase-like protein** | **1,91** |
| **LmjF13.1630** | **mitochondrial DNA polymerase I protein D, putative** | **1,80** |
| **LmjF13.1690** | **hypothetical protein, conserved** | **2,96** |
| LmjF14.1340 | delta-6 fatty acid desaturase, putative | 2,16 |
| LmjF14.1480 | glutathione-S-transferase/glutaredoxin,putative | 1,89 |
| **LmjF15.0230** | **lysyl-tRNA synthetase, putative** | **2,84** |
| **LmjF15.0270** | **replication Factor A 28 kDa subunit, putative** | **1,86** |
| **LmjF15.0520** | **ecotin, putative** | **2,26** |
| LmjF15.1010 | glutamate dehydrogenase | 2,14 |
| LmjF15.1240 | nucleoside transporter 1, putative | 1,87 |
| **LmjF15.1350** | **hypothetical protein, conserved** | **1,74** |
| **LmjF15.1470** | **ribosomal protein S6, putative** | **2,40** |
| **LmjF15.1520** | **hypothetical protein, conserved** | **2,38** |
| **LmjF16.0420** | **hypothetical protein, conserved** | **2,21** |
| **LmjF16.0520** | **hypothetical protein, conserved** | **1,94** |
| LmjF17.0086 | elongation factor 1-alpha | 1,80 |
| **LmjF17.0340** | **hypothetical protein, conserved** | **1,81** |
| **LmjF17.0390** | **protein kinase, putative** | **2,00** |
| **LmjF17.0430** | **hypothetical protein, conserved** | **2,61** |
| LmjF17.0550 | RNA-binding protein, putative | 2,04 |
| LmjF17.0570 | hypothetical protein, conserved | 1,84 |
| **LmjF17.0690** | **hypothetical protein, conserved** | **2,26** |
| LmjF17.0780 | hypothetical protein, conserved | 2,13 |
| **LmjF17.0790** | **protein kinase, putative** | **2,09** |
| LmjF17.0980 | hypothetical protein, conserved | 2,12 |
| LmjF17.1250 | hypothetical protein, conserved | 2,02 |
| LmjF17.1290 | translation initiation factor, putative | 1,83 |
| LmjF17.1320 | hypothetical protein, conserved | 1,68 |
| LmjF17.1410 | zinc-finger protein ZPR1, putative | 2,03 |
| LmjF17.1460 | ferrochelatase-like protein | 1,72 |
| **LmjF18.0020** | **diphosphomevalonate decarboxylase, putative** | **2,53** |
| **LmjF18.0190** | **hypothetical protein, unknown function** | **2,00** |
| **LmjF18.0280** | **hypothetical protein, conserved** | **1,73** |
| **LmjF18.0300** | **hypothetical protein, conserved** | **2,46** |
| **LmjF18.0430** | **hypothetical protein, unknown function** | **2,03** |
| **LmjF18.1060** | **calpain-like cysteine peptidase, putative** | **2,60** |
| **LmjF19.0060** | **40S ribosomal protein S2** | **1,71** |
| **LmjF19.0100** | **fibrillarin, putative** | **1,89** |
| **LmjF19.0240** | **hypothetical protein, conserved** | **2,20** |
| **LmjF19.0800** | **ABC transporter, putative** | **2,35** |
| **LmjF20.0030** | **hypothetical protein, conserved** | **2,29** |
| **LmjF20.0050** | **anti-silencing protein asf 1-like protein** | **1,85** |
| **LmjF20.0140** | **hypothetical protein, conserved** | **1,93** |
| **LmjF20.0460** | **hypothetical protein, conserved** | **1,85** |
| **LmjF20.0700** | **hypothetical protein, conserved** | **2,07** |
| LmjF20.0705 | hypothetical protein, conserved | 1,92 |
| LmjF20.1050 | hypothetical protein, unknown function | 1,87 |
| **LmjF20.1070** | **hypothetical protein, unknown function** | **1,96** |
| **LmjF20.1670** | **ribosome biogenesis protein, putative** | **1,76** |
| **LmjF20.1700** | **hypothetical protein, conserved** | **2,14** |
| **LmjF21.0440** | **ubiquitin-conjugating enzyme-like protein** | **2,75** |
| LmjF21.0450 | hypothetical protein, unknown function | 1,99 |
| **LmjF21.0540** | **la RNA binding protein, putative** | **2,24** |
| **LmjF21.0710** | **ribonuclease L inhibitor, putative** | **2,50** |
| LmjF21.0883 | hypothetical protein, conserved | 2,03 |
| **LmjF21.1090** | **t-complex protein 1, delta subunit, putative** | **2,27** |
| LmjF21.1240 | hypothetical protein, conserved | 1,88 |
| LmjF21.1550 | 40S ribosomal protein S11, putative | 2,18 |
| **LmjF21.1560** | **hypothetical protein, conserved** | **1,84** |
| **LmjF21.1730** | **hypothetical protein, conserved** | **1,79** |
| **LmjF22.0170** | **hypothetical protein, conserved** | **2,31** |
| **LmjF22.0225** | **hypothetical protein, conserved** | **2,24** |
| **LmjF22.0600** | **hypothetical protein, conserved** | **2,05** |
| **LmjF22.0790** | **hypothetical protein, conserved** | **1,74** |
| LmjF22.1360 | farnesyl pyrophosphate synthase, putative | 3,08 |
| **LmjF23.0010** | **hypothetical protein, conserved** | **1,74** |
| **LmjF23.0030** | **beta propeller protein, putative** | **3,13** |
| **LmjF23.0040** | **peroxidoxin** | **2,66** |
| **LmjF23.0050** | **cyclophilin, putative** | **3,30** |
| **LmjF23.0090** | **hypothetical protein, conserved** | **1,76** |
| **LmjF23.0130** | **vacuolar type h+ ATPase subunit, putative** | **2,07** |
| **LmjF23.0200** | **endoribonuclease L-PSP (pb5), putative** | **4,09** |
| **LmjF23.0230** | **hypothetical protein, conserved** | **1,94** |
| **LmjF23.0240** | **terbinafine resistance locus protein (yip1)** | **2,25** |
| **LmjF23.0250** | **multidrug resistance protein, putative** | **2,70** |
| **LmjF23.0330** | **hypothetical protein, conserved** | **1,78** |
| **LmjF23.0340** | **(H+)-ATPase G subunit, putative** | **2,28** |
| **LmjF23.0370** | **hypothetical protein, conserved** | **1,90** |
| **LmjF23.0380** | **ABC transporter-like protein** | **2,05** |
| **LmjF23.0510** | **hypothetical protein, conserved** | **2,76** |
| **LmjF23.0540** | **acetyl-CoA synthetase, putative** | **1,87** |
| LmjF23.0550 | ubiquitin-activating enzyme E1, putative | 1,86 |
| LmjF23.0560 | kinesin, putative | 2,37 |
| LmjF23.0580 | hypothetical protein, conserved | 1,96 |
| LmjF23.0620 | hypothetical protein, conserved | 2,40 |
| LmjF23.0680 | hypothetical protein, conserved | 1,96 |
| LmjF23.0690 | 3-ketoacyl-coa thiolase-like protein | 1,93 |
| LmjF23.0700 | hypothetical protein, conserved | 2,94 |
| LmjF23.0720 | hypothetical protein, unknown function | 1,75 |
| LmjF23.0740 | hypothetical protein, conserved | 1,91 |
| LmjF23.0760 | mitochondrial RNA binding protein, putative | 2,42 |
| **LmjF23.0820** | **hypothetical protein, conserved** | **2,68** |
| LmjF23.0830 | Na/H antiporter-like protein | 2,16 |
| LmjF23.0840 | hypothetical protein, unknown function | 1,70 |
| LmjF23.0870 | beta-fructosidase-like protein | 1,77 |
| LmjF23.0880 | beta-fructosidase-like protein | 1,73 |
| LmjF23.0990 | hypothetical protein, conserved | 2,29 |
| LmjF23.1000 | hypothetical protein, unknown function | 1,82 |
| LmjF23.1010 | hypothetical protein, conserved | 1,82 |
| LmjF23.1025 | hypothetical protein, conserved | 1,82 |
| LmjF23.1155 | hypothetical protein, unknown function | 1,98 |
| LmjF23.1165 | coronin, putative | 2,12 |
| LmjF23.1220 | t-complex protein 1, gamma subunit, putative | 2,63 |
| LmjF23.1240 | hypothetical protein, conserved | 1,86 |
| LmjF23.1250 | hypothetical protein, conserved | 2,31 |
| LmjF23.1260 | hypothetical protein, conserved | 1,75 |
| LmjF23.1263 | hypothetical protein, conserved | 2,27 |
| LmjF23.1267 | hypothetical protein, unknown function | 2,00 |
| LmjF23.1290 | hypothetical protein, unknown function | 1,97 |
| LmjF23.1300 | lathosterol oxidase-like protein | 1,92 |
| LmjF23.1340 | hypothetical protein, conserved | 1,74 |
| LmjF23.1350 | acetyltransferase-like protein | 2,07 |
| LmjF23.1400 | Ran-binding protein, putative | 1,72 |
| LmjF23.1460 | hypothetical protein, conserved | 1,72 |
| LmjF23.1480 | hypothetical protein, conserved | 1,76 |
| LmjF23.1490 | hypothetical protein, unknown function | 2,03 |
| LmjF23.1520 | protoheme IX farnesyltransferase, putative | 2,02 |
| LmjF23.1580 | hypothetical protein, conserved | 3,31 |
| LmjF23.1650 | hypothetical protein | 2,07 |
| LmjF23.1650 | hypothetical protein | 1,84 |
| LmjF23.1665 | hypothetical protein | 2,18 |
| LmjF23.1700 | hypothetical protein, conserved | 1,78 |
| LmjF23.1710 | hypothetical protein, conserved | 2,09 |
| LmjF23.1720 | hypothetical protein, conserved | 1,84 |
| LmjF23.1750 | hypothetical protein, conserved | 1,98 |
| LmjF23.1770 | Csl4p homologue, putative | 1,78 |
| **LmjF24.0480** | **hypothetical predicted Kelch-domain protein** | **1,83** |
| **LmjF24.0720** | **mitochondrial translocase subunit, putative** | **1,74** |
| **LmjF24.0760** | **DNA repair and recombination protein RAD54, putative** | **1,77** |
| **LmjF24.1400** | **hypothetical protein, conserved** | **1,80** |
| LmjF24.1540 | adaptor complex AP-3 small subunit, putative | 1,69 |
| **LmjF24.1910** | **hypothetical protein, conserved** | **2,45** |
| LmjF24.2110 | hypothetical protein, conserved | 2,03 |
| LmjF25.0620 | RNA polymerase I second largest subunit, putative | 1,68 |
| **LmjF25.0920** | **hypothetical protein, conserved** | **1,93** |
| LmjF25.1120 | aldehyde dehydrogenase, mitochondrial precursor | 1,74 |
| **LmjF25.1470** | **cyclin** | **1,89** |
| LmjF26.0770 | hypothetical protein, unknown function | 1,88 |
| **LmjF26.0970** | **hypothetical protein, conserved** | **2,25** |
| **LmjF26.1190** | **hypothetical protein, conserved** | **2,09** |
| LmjF26.1590 | hypothetical protein, unknown function | 1,90 |
| **LmjF26.1910** | **hypothetical protein, conserved** | **1,80** |
| **LmjF26.2630** | **hypothetical protein, conserved** | **1,93** |
| **LmjF27.0050** | **DEAD-box helicase-like protein** | **2,23** |
| LmjF27.0060 | hypothetical protein, conserved | 1,73 |
| LmjF27.0070 | hypothetical protein, conserved | 1,80 |
| **LmjF27.0130** | **hypothetical protein, conserved** | **3,01** |
| **LmjF27.0210** | **hypothetical protein, conserved** | **1,77** |
| **LmjF27.0350** | **hypothetical protein, conserved** | **2,55** |
| **LmjF27.0430** | **hypothetical protein, conserved** | **2,18** |
| LmjF27.0650 | hypothetical protein, conserved | 1,79 |
| **LmjF27.0700** | **hypothetical protein, conserved** | **2,38** |
| **LmjF27.0760** | **small GTP-binding protein Rab1, putative** | **2,02** |
| **LmjF27.0870** | **hypothetical protein, conserved** | **1,77** |
| LmjF27.0930 | isovaleryl-coA dehydrogenase, putative | 1,96 |
| **LmjF27.0980** | **ABC transporter, putative** | **1,79** |
| **LmjF27.1050** | **vesicular transport protein (CDC48 homologue), putative** | **1,77** |
| **LmjF27.1080** | **hypothetical protein, conserved** | **1,96** |
| **LmjF27.1120** | **hypothetical protein, conserved** | **2,32** |
| **LmjF27.1160** | **hypothetical protein, conserved** | **1,91** |
| **LmjF27.1250** | **hypothetical protein, conserved** | **2,04** |
| **LmjF27.1265** | **cation transporter, putative** | **2,09** |
| LmjF27.1275 | hypothetical protein, conserved | 1,71 |
| **LmjF27.1300** | **hypothetical protein, conserved** | **2,22** |
| LmjF27.1310 | arginyl-tRNA synthetase, putative | 1,78 |
| LmjF27.1390 | 60S acidic ribosomal subunit protein, putative | 2,87 |
| **LmjF27.1450** | **hypothetical protein, conserved** | **2,00** |
| **LmjF27.1460** | **proteasome regulatory non-ATP-ase subunit 3, putative** | **1,72** |
| **LmjF27.1580** | **amino acid transporter, putative** | **2,23** |
| **LmjF27.1680** | **hypothetical protein, conserved** | **1,69** |
| LmjF27.1690 | hypothetical protein, conserved | 1,85 |
| LmjF27.1710 | eukaryotic translation release factor, putative | 1,83 |
| **LmjF27.1810** | **glycosomal phosphoenolpyruvate carboxykinase, putative** | **2,06** |
| **LmjF27.1870** | **trypanothione synthetase, putative** | **2,06** |
| **LmjF27.1980** | **FtsJ cell division protein, putative** | **2,11** |
| LmjF27.2000 | hypothetical protein, conserved | 1,72 |
| **LmjF27.2010** | **hypothetical protein, conserved** | **1,98** |
| **LmjF27.2110** | **hypothetical protein, unknown function** | **1,89** |
| **LmjF27.2270** | **hypothetical protein, conserved** | **2,12** |
| **LmjF27.2300** | **transcription elongation regulator-like protein** | **1,85** |
| **LmjF27.2320** | **protein phosphatase-like protein** | **2,06** |
| **LmjF27.2350** | **vesicle-associated membrane protein (VAMP), putative** | **2,34** |
| **LmjF28.0040** | **hypothetical protein, conserved** | **2,05** |
| LmjF28.0520 | protein kinase, putative | 1,75 |
| **LmjF28.0550** | **RAD51 protein, putative** | **1,81** |
| **LmjF28.0890** | **ribonucleoside-diphosphate reductase large chain, putative** | **2,45** |
| **LmjF28.1060** | **hypothetical protein, conserved** | **3,09** |
| **LmjF28.1260** | **hypothetical protein, conserved** | **1,95** |
| **LmjF28.1310** | **ATP-dependent RNA helicase, putative** | **2,12** |
| **LmjF28.1740** | **hypothetical protein, conserved** | **1,87** |
| **LmjF28.1810** | **hypothetical protein, conserved** | **2,04** |
| **LmjF28.1820** | **replication factor A, 51kDa subunit, putative** | **2,03** |
| LmjF28.1860 | hypothetical protein, conserved | 1,74 |
| LmjF28.1880 | hypothetical protein, conserved | 1,72 |
| **LmjF28.1940** | **hypothetical protein, conserved** | **1,77** |
| **LmjF28.2060** | **DNA-directed RNA polymerase-like protein** | **1,69** |
| LmjF28.2560 | 40S ribosomal protein S17, putative | 1,97 |
| **LmjF29.0250** | **oxidase-like protein** | **2,41** |
| **LmjF29.0830** | **hypothetical protein, conserved** | **2,21** |
| **LmjF29.0850** | **high mobility group protein homolog tdp-1, putative** | **2,32** |
| **LmjF29.0865** | **hypothetical protein, conserved** | **1,86** |
| **LmjF29.0890** | **hypothetical protein, conserved** | **1,82** |
| LmjF29.1250 | hypothetical protein, unknown function | 2,12 |
| **LmjF29.1260** | **hypothetical protein, conserved** | **1,74** |
| LmjF29.1380 | hypothetical protein, conserved | 2,16 |
| LmjF29.2350 | hypothetical protein, conserved | 1,77 |
| **LmjF29.2510** | **6-phospho-1-fructokinase, putative** | **1,86** |
| LmjF30.0500 | hypothetical protein, conserved | 1,75 |
| **LmjF30.0620** | **ribosome biogenesis regulatory protein (RRS1), putative** | **1,83** |
| LmjF30.0790 | hypothetical protein, conserved | 1,79 |
| **LmjF30.1030** | **DnaJ-like protein** | **1,95** |
| **LmjF30.1290** | **U3 small nuclear ribonucloprotein (snRNP), putative** | **2,04** |
| LmjF30.1590 | hypothetical protein, conserved | 1,79 |
| **LmjF30.1730** | **hypothetical protein, conserved** | **2,27** |
| LmjF30.1790 | DnaJ domain protein, putative | 1,77 |
| LmjF30.2160 | hypothetical protein, conserved | 1,71 |
| **LmjF30.2200** | **hypothetical protein, conserved** | **1,75** |
| LmjF30.2330 | hypothetical protein, unknown function | 1,87 |
| **LmjF30.2580** | **reticulon domain protein, 22 kDa potentially aggravating protein (paple22)** | **1,90** |
| **LmjF30.2900** | **aldehyde dehydrogenase, putative** | **1,89** |
| LmjF30.3090 | hypothetical protein, conserved | 1,90 |
| **LmjF30.3140** | **hypothetical protein, conserved** | **1,78** |
| **LmjF30.3440** | **DNA ligase I, putative** | **1,85** |
| **LmjF30.3550** | **cytochrome p450-like protein** | **1,73** |
| LmjF30.3570 | zinc finger-domain protein, putative | 1,98 |
| **LmjF31.0080** | **hypothetical protein, conserved** | **2,14** |
| LmjF31.0200 | hypothetical protein, conserved | 1,76 |
| **LmjF31.0340** | **amino acid transporter aATP11, putative** | **1,76** |
| **LmjF31.0470** | **hypothetical protein, conserved** | **1,84** |
| **LmjF31.0540** | **hypothetical protein, conserved** | **1,84** |
| LmjF31.0570 | amino acid transporter aATP11, putative | 1,73 |
| **LmjF31.0740** | **hypothetical protein, conserved** | **2,12** |
| **LmjF31.0800** | **hypothetical protein, conserved** | **1,75** |
| LmjF31.0880 | amino acid transporter, putative | 1,73 |
| **LmjF31.1010** | **hypothetical protein, conserved** | **1,73** |
| **LmjF31.1220** | **vacuolar-type proton translocating pyrophosphatase 1, putative** | **2,65** |
| **LmjF31.1610** | **diphthine synthase-like protein** | **1,75** |
| LmjF31.2000 | GP63-like protein, leishmanolysin-like protein | 2,14 |
| **LmjF31.2090** | **hypothetical protein, unknown function** | **1,79** |
| **LmjF31.2270** | **hypothetical protein, conserved** | **1,78** |
| **LmjF31.2310** | **3'-nucleotidase/nuclease precursor, putative** | **2,93** |
| **LmjF31.2450** | **hypothetical protein, unknown function** | **3,61** |
| **LmjF31.2600** | **calreticulin, putative** | **1,75** |
| **LmjF31.2760** | **kinetoplast-associated protein-like protein** | **1,84** |
| LmjF31.2770 | hypothetical protein, unknown function | 1,77 |
| **LmjF31.2785** | **hypothetical protein, conserved** | **2,59** |
| **LmjF31.2860** | **protein kinase, putative** | **1,98** |
| **LmjF31.3130** | **methylcrotonoyl-coa carboxylase biotinylated subunitprotein-like protein** | **1,70** |
| LmjF32.0030 | hypothetical protein, unknown function | 1,78 |
| LmjF32.0150 | hypothetical protein, conserved | 1,82 |
| LmjF32.0210 | hypothetical protein, conserved | 1,95 |
| LmjF32.0230 | dynein light chain, flagellar outer arm, putative | 1,90 |
| **LmjF32.0240** | **hypothetical protein, conserved** | **2,05** |
| **LmjF32.0450** | **40S ribosomal protein S2** | **1,83** |
| **LmjF32.0510** | **hypothetical protein, conserved** | **1,91** |
| LmjF32.0570 | ATP-dependent RNA helicase-like protein, putative | 1,73 |
| **LmjF32.0700** | **ubiquitin carrier protein 4, putative** | **2,28** |
| LmjF32.0830 | hypothetical protein, unknown function | 1,86 |
| LmjF32.0870 | phenylalanyl-tRNA synthetase alpha chain, putative | 1,71 |
| LmjF32.0890 | ethanolamine-phosphate cytidylyltransferase, putative | 1,91 |
| LmjF32.1110 | mitochondrial carrier protein, putative | 1,84 |
| LmjF32.1130 | hypothetical protein, conserved | 1,69 |
| LmjF32.1490 | hypothetical protein, conserved | 2,04 |
| LmjF32.1570 | hypothetical protein, conserved | 1,73 |
| **LmjF32.1600** | **hypothetical protein, conserved** | **2,01** |
| LmjF32.1640 | hypothetical protein, conserved | 2,10 |
| LmjF32.1650 | hypothetical protein, conserved | 1,80 |
| LmjF32.1660 | hypothetical protein, conserved | 1,69 |
| **LmjF32.1700** | **hypothetical protein, conserved** | **2,04** |
| LmjF32.1740 | zinc finger protein 2, putative | 1,86 |
| LmjF32.1750 | hypothetical protein, conserved | 1,76 |
| LmjF32.1830 | iron superoxide dismutase, putative | 1,89 |
| LmjF32.1900 | hypothetical protein, conserved | 1,94 |
| LmjF32.1990 | hypothetical protein, conserved | 1,92 |
| LmjF32.2120 | hypothetical protein, conserved | 1,92 |
| **LmjF32.2150** | **hypothetical protein, conserved** | **1,95** |
| **LmjF32.2155** | **hypothetical protein, conserved** | **2,26** |
| LmjF32.2180 | hypothetical protein, conserved | 1,80 |
| LmjF32.2310 | hypothetical protein, conserved | 1,89 |
| **LmjF32.2520** | **hypothetical protein, unknown function** | **2,17** |
| LmjF32.2610 | hypothetical protein, conserved | 1,80 |
| LmjF32.2630 | superoxide dismutase, putative | 1,95 |
| **LmjF32.2640** | **cystathionine beta-lyase, putative** | **2,68** |
| LmjF32.2910 | ubiquitin hydrolase, putative | 1,97 |
| **LmjF32.2930** | **tubulin-tyrosine ligase-like protein** | **2,56** |
| **LmjF32.2940** | **hypothetical protein, conserved** | **2,47** |
| LmjF32.3010 | hypothetical protein, conserved | 2,00 |
| LmjF32.3050 | hypothetical protein, conserved | 1,87 |
| LmjF32.3060 | hypothetical protein, conserved | 1,79 |
| LmjF32.3270 | chaperonin alpha subunit, putative | 1,71 |
| LmjF32.3780 | kinetoplast DNA-associated protein, putative | 2,17 |
| LmjF32.3890 | AUT2/APG4/ATG4 cysteine peptidase, putative | 1,89 |
| LmjF32.3980 | hypothetical protein, conserved | 2,07 |
| **LmjF33.0920** | **40S ribosomal protein S3, putative** | **1,74** |
| LmjF33.0960 | nicotinate phosphoribosyltransferase, putative | 1,70 |
| **LmjF33.1140** | **hypothetical protein, conserved** | **4,32** |
| **LmjF33.1350** | **hypothetical protein, conserved** | **2,16** |
| **LmjF33.1450** | **hypothetical protein, conserved** | **1,96** |
| **LmjF33.1590** | **hypothetical protein, conserved** | **2,84** |
| **LmjF33.1930** | **phosphoribosylpyrophosphate synthetase, putative** | **2,34** |
| **LmjF33.3160** | **hypothetical protein, conserved** | **3,21** |
| **LmjF33.3240** | **h1 histone-like protein** | **1,99** |
| **LmjF34.0010** | **hypothetical protein, conserved** | **2,22** |
| **LmjF34.0070** | **ascorbate-dependent peroxidase, putative** | **2,38** |
| LmjF34.0380 | hypothetical protein, unknown function | 1,71 |
| **LmjF34.0390** | **hypothetical protein, unknown function** | **2,22** |
| **LmjF34.0510** | **phosphoglycan beta 1,2 arabinosyltransferase** | **2,02** |
| LmjF34.1410 | d-isomer specific 2-hydroxyacid dehydrogenase-like protein | 1,93 |
| **LmjF34.2050** | **ATP-dependent RNA helicase, putative** | **1,93** |
| LmjF34.2740 | hypothetical protein, unknown function | 1,70 |
| **LmjF34.4610** | **hypothetical protein, conserved** | **1,71** |
| LmjF35.0270 | hypothetical protein, conserved | 1,90 |
| LmjF35.0600 | 60S ribosomal protein L18a, putative | 2,26 |
| **LmjF35.0880** | **hypothetical protein, conserved** | **1,91** |
| LmjF35.1030 | hypothetical protein, unknown function | 1,69 |
| **LmjF35.1040** | **hypothetical protein, conserved** | **1,74** |
| LmjF35.1230 | short chain dehydrogenase, putative | 1,89 |
| **LmjF35.1440** | **60S ribosomal protein L2, putative** | **1,76** |
| **LmjF35.1700** | **RNA 3'-terminal phosphate cyclase, putative** | **1,99** |
| LmjF35.1800 | hypothetical protein, conserved | 1,72 |
| LmjF35.2010 | 40S ribosomal protein S6, putative | 1,72 |
| **LmjF35.2080** | **calcium motive p-type ATPase, putative** | **1,78** |
| **LmjF35.2750** | **hypothetical protein, conserved** | **2,05** |
| **LmjF35.3680** | **ribulose-phosphate 3-epimerase, putative** | **2,28** |
| **LmjF35.4470** | **hypothetical protein, conserved** | **2,08** |
| **LmjF35.4700** | **MCAK-like kinesin, putative** | **1,88** |
| **LmjF35.4760** | **hypothetical protein, conserved** | **1,76** |
| LmjF35.4770 | peptidyl-prolyl cis-trans isomerase (cyclophilin-40), putative | 1,70 |
| **LmjF35.5040** | **polyadenylate-binding protein 1, putative** | **3,49** |
| LmjF35.5090 | hypothetical protein, conserved | 1,93 |
| **LmjF35.5100** | **60S ribosomal protein L37** | **1,98** |
| **LmjF35.5200** | **hypothetical protein, conserved** | **2,23** |
| LmjF35.5330 | isopentenyl-diphosphate delta-isomerase, putative | 1,78 |
| LmjF36.0330 | ribonuclease HII, putative | 1,70 |
| **LmjF36.0550** | **cell division related protein kinase 2** | **1,98** |
| **LmjF36.0600** | **ubiquitin/ribosomal protein S27a, putative** | **1,72** |
| **LmjF36.0660** | **hypothetical protein, conserved** | **2,26** |
| **LmjF36.1160** | **hypothetical protein, conserved** | **1,80** |
| **LmjF36.1170** | **hypothetical protein, conserved** | **2,11** |
| LmjF36.1260 | fructose-1,6-bisphosphate aldolase | 1,87 |
| **LmjF36.2000** | **hypothetical protein, conserved** | **2,31** |
| **LmjF36.2035** | **hypothetical protein, conserved** | **1,93** |
| **LmjF36.2360** | **tyrosine aminotransferase, putative** | **2,10** |
| **LmjF36.2590** | **membrane-bound acid phosphatase 2, putative** | **3,46** |
| **LmjF36.3070** | **fibrillarin** | **2,47** |
| **LmjF36.3140** | **hypothetical protein, conserved** | **1,90** |
| **LmjF36.3760** | **60S ribosomal protein L10a, putative** | **1,89** |
| **LmjF36.3910** | **S-adenosylhomocysteine hydrolase** | **1,94** |
| **LmjF36.4580** | **hypothetical protein, conserved** | **1,77** |
| **LmjF36.5365** | **hypothetical protein, conserved** | **1,78** |
| **LmjF36.5820** | **mkiaa0324 protein-like protein** | **1,94** |
| **LmjF36.5845** | **kinetoplast-associated protein, putative** | **2,18** |
| LmjF36.6090 | hypothetical protein, conserved | 1,71 |
| **LmjF36.7000** | **hypothetical protein, conserved** | **2,54** |

GeneDB systematic IDs are related to *L.major* according to microarray labeled probes.

In **Bold**, genes up or downregulated in both Sb^III^2700.2 and Sb^III^2700.3 mutants.
